# Comparative Evaluation of WCLV (1.2344), Uddeholm Unimax, and Uddeholm QRO 90 Supreme Tool Steels for Die Forging †

**DOI:** 10.3390/ma19163526

**Published:** 2026-08-20

**Authors:** Maciej Wąsowicz, Adam Patalas, Artur Meller, Stanisław Legutko, Piotr Siwak, Vit Černohlávek

**Affiliations:** 1 Faculty of Mechanical Engineering, Poznan University of Technology, ul. Jacka Rychlewskiego 1, 61-131 Poznań, Poland; adam.patalas@put.poznan.pl (A.P.); stanislaw.legutko@put.poznan.pl (S.L.); piotr.siwak@put.poznan.pl (P.S.); 2 Fabryka Armatur “Swarzędz” sp. z o.o., ul. Świerkowa 27, Rabowice, 62-020 Swarzędz, Poland; artur.meller@fa-swarzedz.com.pl; 3 Faculty of Mechanical Engineering, J. E. Purkyne University in Usti nad Labem, Pasteurova 3334/7, 400 01 Usti nad Labem, Czech Republic; vit.cernohlavek@ujep.cz

**Keywords:** hot-forming tool steels, hot forging, abrasive wear, tool life, tool wear, tribology

## Abstract

This study presents a comparative evaluation of the wear performance of three hot-work tool steels—WCLV (1.2344), Uddeholm Unimax, and Uddeholm QRO 90 Supreme—for die forging applications. The materials were characterized in terms of hardness and bulk chemical composition using Vickers hardness testing and X-ray fluorescence spectroscopy. Tribological behavior was investigated using ball-on-disc tests, while industrial performance was assessed by analyzing forging punches after the production of 16,250 components. Surface degradation was quantified using optical profilometry and three-dimensional roughness parameters. Measured hardness values were 591 HV for WCLV, 622 HV for QRO 90 Supreme, and 639 HV for Uddeholm Unimax. The average friction coefficients were 0.88, 0.92, and 0.77, respectively. Unimax also exhibited the lowest volumetric wear, reaching 0.04683 mm^3^ (R19 mm) and 0.03384 mm^3^ (R22 mm), compared with 0.08646–0.13095 mm^3^ for WCLV and 0.09598–0.13635 mm^3^ for QRO 90 Supreme. This corresponds to approximately 45–70% lower wear relative to the other steels. Industrial trials confirmed improved surface stability of Unimax punches after service. The observed trends are consistent with differences in alloying content and the expected microstructural response associated with chromium and molybdenum additions. Overall, Uddeholm Unimax demonstrated the most favorable balance of hardness, friction behavior, and wear resistance.

## 1. Introduction

The durability of forging tools is a key factor affecting both production efficiency and product quality in hot forging operations. During service, forging tools are exposed to severe thermo-mechanical conditions, including high contact pressures and cyclic temperature variations. These conditions promote the development of multiple degradation mechanisms, which significantly increase manufacturing costs and may account for up to 40% of the total production expenditure. Consequently, the selection and optimization of tool materials are of fundamental importance for improving wear resistance and extending tool service life [1,2,3,4,5].

Hot forging tools are subjected to cyclic mechanical stresses that can exceed 1000 MPa, combined with rapid heating and cooling cycles. As a result, their degradation is governed by complex wear mechanisms such as abrasive and adhesive wear, thermal fatigue, plastic deformation, and crack initiation. Tool performance is therefore strongly influenced by the intrinsic material properties, including hardness, toughness, and microstructural stability, as well as by external process conditions. Despite ongoing progress in the development of advanced tool steels, the interaction between concurrent wear mechanisms remains insufficiently understood [6,7,8,9,10].

From a materials engineering perspective, the selection of appropriate hot-work tool steels represents one of the most effective strategies for enhancing tool life. Steels such as WCLV, Uddeholm Unimax, and Uddeholm QRO 90 Supreme are widely used due to their favorable combinations of strength, thermal resistance, and toughness. However, material selection must be complemented by optimized heat treatment procedures, particularly quenching and tempering, to achieve a suitable balance between hardness and fracture resistance. In addition, surface engineering approaches—including nitriding, protective coatings, and laser-based treatments—are increasingly employed to modify surface microstructure, improve hardness, and reduce friction. Despite the widespread industrial use of these steels, a direct comparative assessment of WCLV, Uddeholm Unimax, and Uddeholm QRO 90 Supreme under identical laboratory and industrial workflows remains limited in the available literature. Most previous studies focus on individual materials or analyze tool performance under different processing conditions, which makes it difficult to clearly identify the material-related factors governing their relative wear resistance [11,12].

Recent studies indicate that forging tool wear typically results from the combined action of abrasive and adhesive mechanisms, thermo-mechanical fatigue, and localized plastic deformation. For example, WCLV steel has been reported to exhibit increased susceptibility to abrasive wear, whereas Uddeholm Unimax often demonstrates superior performance due to its higher hardness and lower coefficient of friction. Advanced surface modification techniques, such as hybrid coatings and preventive pad welding, have shown potential for further enhancing wear resistance; however, their effectiveness is strongly dependent on tool geometry and process-specific conditions [13,14,15]. Consequently, the relative performance of commonly used hot-work tool steels when subjected to comparable tribological conditions and subsequently validated in industrial forging operations remains insufficiently documented.

In addition to material-related factors, tool life can be significantly improved through optimized tool design and the application of effective lubrication and cooling strategies. Experimental and numerical investigations have demonstrated that precise control of forging parameters, including temperature and lubrication conditions, can substantially reduce wear rates. Moreover, monitoring techniques based on acoustic emission signals have emerged as promising tools for real-time assessment of tool degradation, enabling condition-based maintenance and improved operational reliability [16,17,18,19].

The aim of this study is to evaluate selected approaches for improving the durability of forging tools, with particular emphasis on the role of tool steel selection. By comparing the wear behavior of WCLV, Uddeholm Unimax, and Uddeholm QRO 90 Supreme steels under both laboratory and industrial conditions, this work seeks to contribute to a deeper understanding of material-related factors governing tool wear and to support the development of cost-effective forging solutions.

## 2. Materials and Methods

### 2.1. Material Characterization

The experimental investigations were conducted on three commercially available hot-work tool steels: WCLV (1.2344), Uddeholm Unimax, and Uddeholm QRO 90 Supreme. These materials are commonly applied in hot forging due to their favorable thermo-mechanical properties. All steels were produced using the electroslag remelting (ESR) process to ensure high cleanliness and structural homogeneity. The materials were subsequently subjected to mechanical machining to obtain the required specimen geometry. Heat treatment, including hardening and tempering, was carried out by the official material distributor in accordance with the recommended technological procedures for each grade. Prior to testing, the samples were ground to ensure a consistent surface condition and repeatable tribological measurements.

WCLV steel (1.2344) is an alloyed hot-work tool steel characterized by high hardenability and excellent resistance to tempering. The presence of alloying elements enhances its hardness, oxidation resistance, and tensile and fatigue strength, making it suitable for demanding forging applications [20,21].

Uddeholm QRO 90 Supreme is a premium-grade tool steel designed for operation at elevated temperatures. It exhibits high hot strength, excellent hardness retention at high temperatures, and superior resistance to tempering and thermal fatigue. These properties make it particularly suitable for hot forging tools exposed to severe thermal loading [22].

Uddeholm Unimax steel is distinguished by its very high hardness and strength combined with good ductility. It demonstrates high resistance to abrasive wear and thermal fatigue, as well as excellent hardenability and a high tempering temperature. Owing to this combination of properties, Unimax steel is widely used for hot forging applications [23].

The chemical composition of the investigated steel is summarized in Table 1.

### 2.2. Hardness and Nanoindentation

Hardness measurements were performed using the Vickers hardness testing method. This technique involves pressing a diamond indenter with a pyramidal geometry and an apex angle of 136° into the material surface, followed by measurement of the diagonals of the resulting indentation. The Vickers hardness value is defined as the ratio of the applied load to the surface area of the indentation.

Due to the high hardness of the investigated tool steels, a test load of 5 kgf and a dwell time of 7 s were selected. For each material, five independent hardness measurements were carried out at different locations to ensure statistical reliability [24].

Nanoindentation tests were carried out using a PICODENTOR^®^ HM500 nanoindenter (Helmut Fischer GmbH, Sindelfingen, Germany) equipped with a pyramidal Vickers diamond tip. The experiments were performed at room temperature (23 ± 1 °C) under load-controlled conditions. The loading protocol consisted of 30 s loading, a 5 s dwell period at the maximum load, and 30 s unloading. Maximum loads of 100 mN and 300 mN were applied. For each steel grade and loading condition, ten independent measurements were performed. The Vickers hardness (HV) and indentation elastic modulus (EIT) were determined from the load–displacement curves, with the elastic modulus calculated according to the Oliver–Pharr method from the unloading segment of the curve. Mean values and standard deviations were calculated from ten measurements.

### 2.3. Chemical Composition Measurements

The chemical composition of the investigated steels was determined using X-ray fluorescence (XRF) spectroscopy, which is a non-destructive analytical technique. A Fisher XDV SDD spectrometer (Helmut Fischer GmbH, Sindelfingen, Germany) was employed for this purpose. During the measurement, the samples were irradiated with X-rays generated by an X-ray tube, inducing the emission of characteristic fluorescent radiation from the constituent elements. By analyzing the energy of the emitted radiation, the elemental composition of the material was quantified. For each steel grade, ten measurements were conducted at different positions on the sample surface, focusing on the content of Fe, Cr, Ni, Mn, Mo, and V [25].

### 2.4. Tribological Testing

Tribological behavior was evaluated using ball-on-disc wear tests. The test specimens were disc-shaped samples with dimensions of Ø69 × 5 mm. Prior to testing, the discs were ground to ensure a uniform surface finish and subsequently cleaned with acetone to remove contaminants.

**Figure 1:**
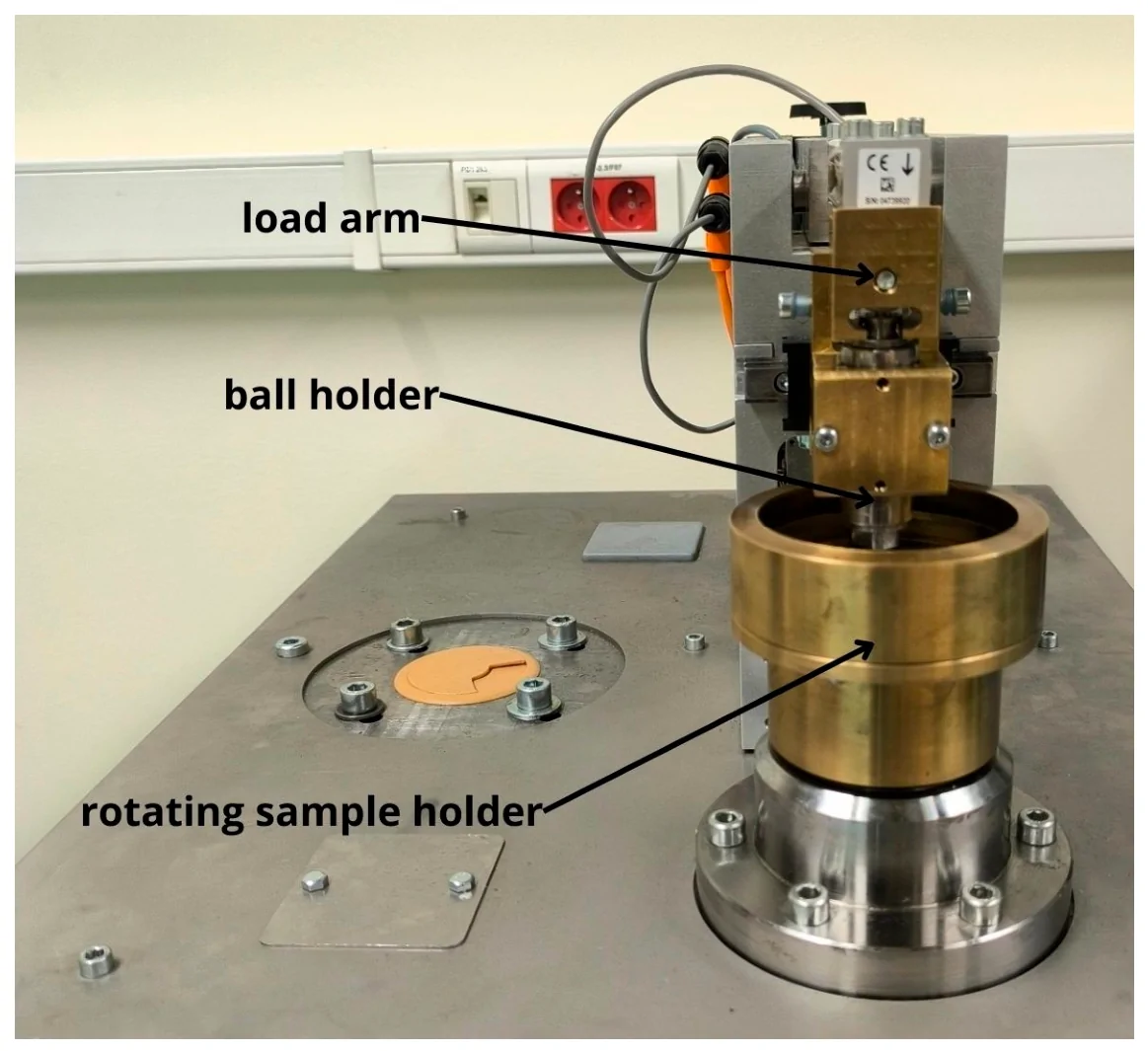
Experimental setup of the ball-on-disc tribological test with the main components of the tribometer indicated.

During the test, a ball made of bearing steel was pressed against the rotating disc under a constant normal load, inducing intensive wear. Figure 1 presents the experimental setup of the ball-on-disc tribological test, with the main components of the tribometer highlighted. Two tests were performed for each material under a normal load of 10 N and a rotational speed of 100 rpm over a test duration of 10 min. The tests differed in friction path length and linear sliding speed due to variations in the contact radius of the ball.

**Figure 2:**
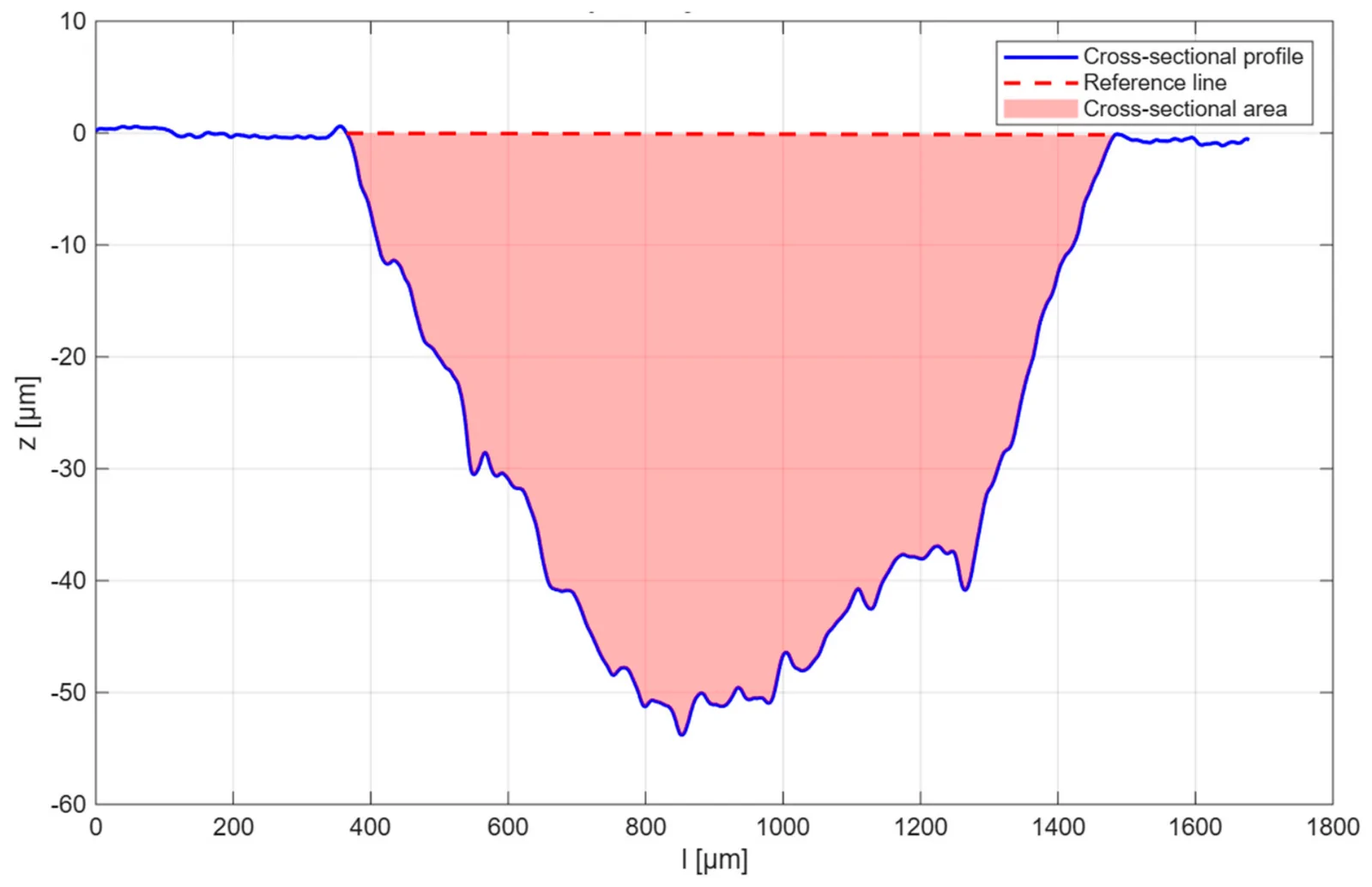
Representative cross-sectional profile of the wear track used for determination of the cross-sectional area and volumetric wear calculations.

Throughout the experiment, the tribometer continuously recorded the normal and friction forces, enabling real-time determination of the coefficient of friction as a function of time [26]. After testing, the volumetric wear of the samples was quantified using an optical profilometer (Alicona portable RL, Alicona GmbH, Raaba, Austria) by analyzing the geometry of the wear tracks. A representative cross-sectional profile of the wear track used for the calculations is shown in Figure 2.

The cross-sectional area of the wear track was determined from profiles obtained using the optical profilometer. Prior to the analysis, the original surface level was established based on the unworn regions adjacent to the wear track and used as a reference line for all calculations. The wear profile was discretized into measurement points spaced at 2 µm intervals. The cross-sectional area was then calculated by numerical integration using the trapezoidal method according to Equation (1), where the area between each pair of neighboring points and the reference line was approximated by a trapezoid with a width of 2 µm. The total cross-sectional area of the wear track was obtained as the sum of the areas of all trapezoids along the profile [27].

(1) $$A=\sum_{i=1}^{n-1}\frac{\left(h_{i}+h_{i+1}\right)}{2}∆x$$

where

*A*—cross-sectional area of the wear track;

*h_i_*—depth of measurement points relative to the reference surface;

Δ*x*—distance between neighboring measurement points (2 µm).

To account for local variations in wear morphology, cross-sectional measurements were performed at three different locations along each wear track, and the reported value corresponds to the mean cross-sectional area. The volumetric wear was subsequently calculated as the product of the mean cross-sectional area and the total wear track length, corresponding to the circumference of the sliding path, according to Equation (2).

(2) $$V=A_{avg}\times 2\pi R\;[{mm}^{3}]$$

where

*V*—volumetric wear [mm^3^];

*A_avg_*—average cross-sectional area of the wear track [mm^2^];

*R*—sliding radius [mm].

### 2.5. Industrial Forging Tests and Surface Analysis

In addition to laboratory-scale testing, wear behavior was evaluated under industrial hot forging conditions. For this purpose, forging punches were manufactured from the investigated steels and used to produce a batch of 16,250 components. The forged material was brass CW617N, processed in a single-operation, flashless forging process. The billets were heated to approximately 680 °C prior to deformation. Forging was performed on a hydraulic press using a sliding tool system equipped with lateral punches. The press cycle time was approximately 4 s.

A liquid graphite-based lubricant was applied during the process. The lubricant was delivered through spray nozzles directed at the punch face and additionally supplied through dedicated channels in the lower die. This lubrication strategy was intended to reduce friction and thermal loading under industrial operating conditions.

Surface topography data were obtained to determine selected surface roughness parameters in accordance with ISO 25178-2:2021 [28], including the arithmetic mean height (S_a_), root mean square height (S_q_), ten-point height (S_10z_), kurtosis (S_ku_), core material volume (V_mc_), and core void volume (V_vc_). The analysis was performed using an elementary sampling length of 250 µm. Prior to parameter calculation, the measured surfaces were processed using a Gaussian filter for inclined planar surfaces in accordance with ISO 16610-61:2015 [29] in order to remove form and waviness components. Additionally, material ratio curves and material distribution histograms were generated to provide further insight into surface layer degradation mechanisms.

## 3. Results and Discussion

### 3.1. Hardness Measurements

**Figure 3:**
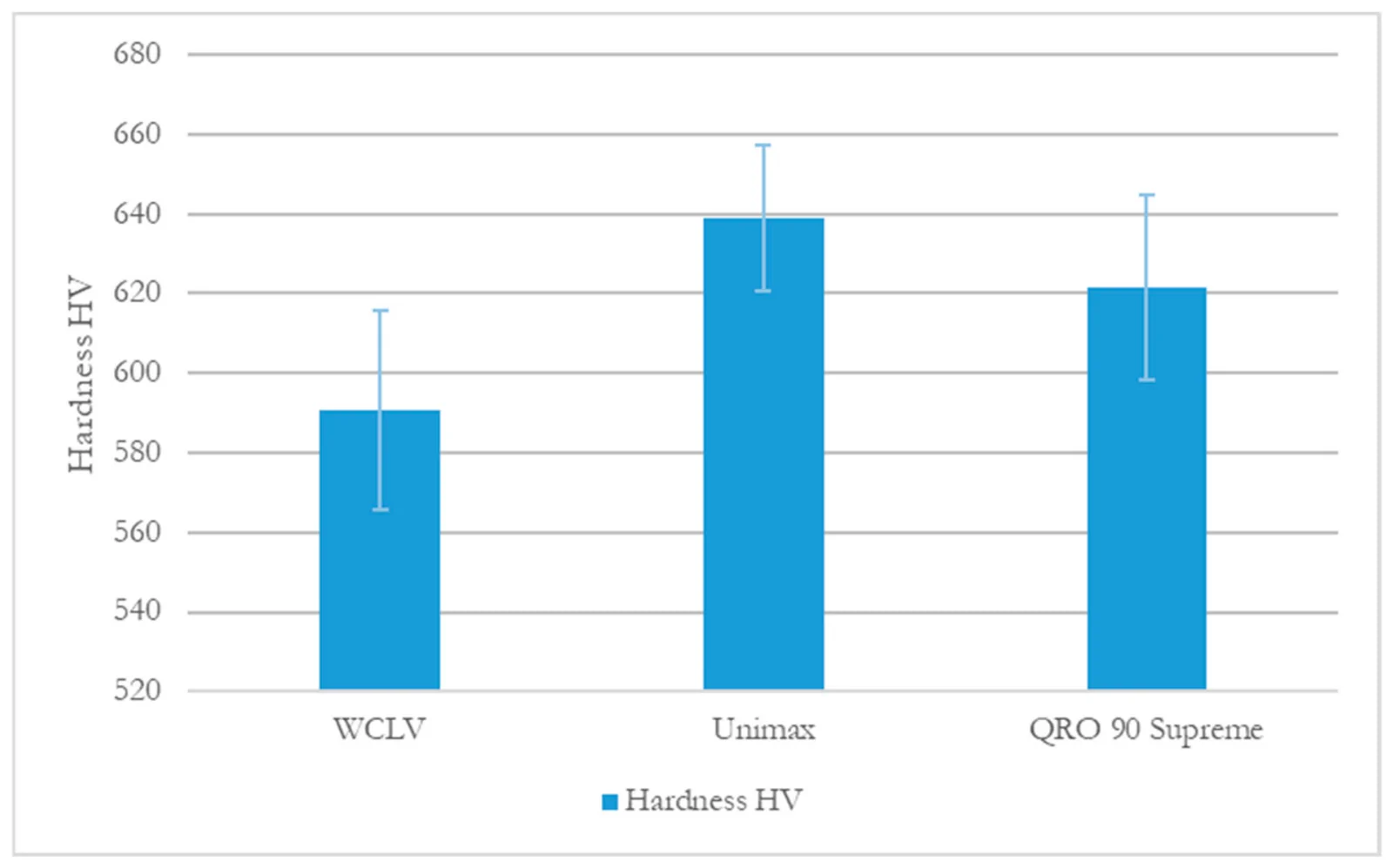
Comparison of the hardness values of the investigated materials [1].

The results of the Vickers hardness measurements for the investigated tool steels are presented in Figure 3. The diagram illustrates the mean hardness values together with 95% confidence intervals. All examined materials exhibited high hardness exceeding 580 HV, which is characteristic of hot-work tool steels after quenching and tempering.

Among the tested materials, WCLV steel showed the lowest average hardness, amounting to 590.66 HV (SD = 8.65 HV). A higher hardness level was recorded for Uddeholm QRO 90 Supreme steel, reaching 621.58 HV (SD = 5.97 HV). The highest hardness was observed for Uddeholm Unimax steel, with an average value of 639.06 HV (SD = 9.28 HV).

Although increased hardness may contribute to improved resistance to abrasive material removal, the performance of hot-forging tools is governed by a combination of properties, including toughness, resistance to thermal fatigue, softening resistance at elevated temperatures, and oxidation behavior. Therefore, hardness differences alone do not fully determine the in-service wear performance of the investigated steels.

### 3.2. Nanoindentation Measurements

The nanoindentation measurements were performed to evaluate the local mechanical properties of the investigated tool steels under low-load conditions. The Vickers hardness (HV) and indentation elastic modulus (EIT) were determined for maximum loads of 100 mN and 300 mN. The results are presented as mean values with corresponding standard deviations.

**Figure 4:**
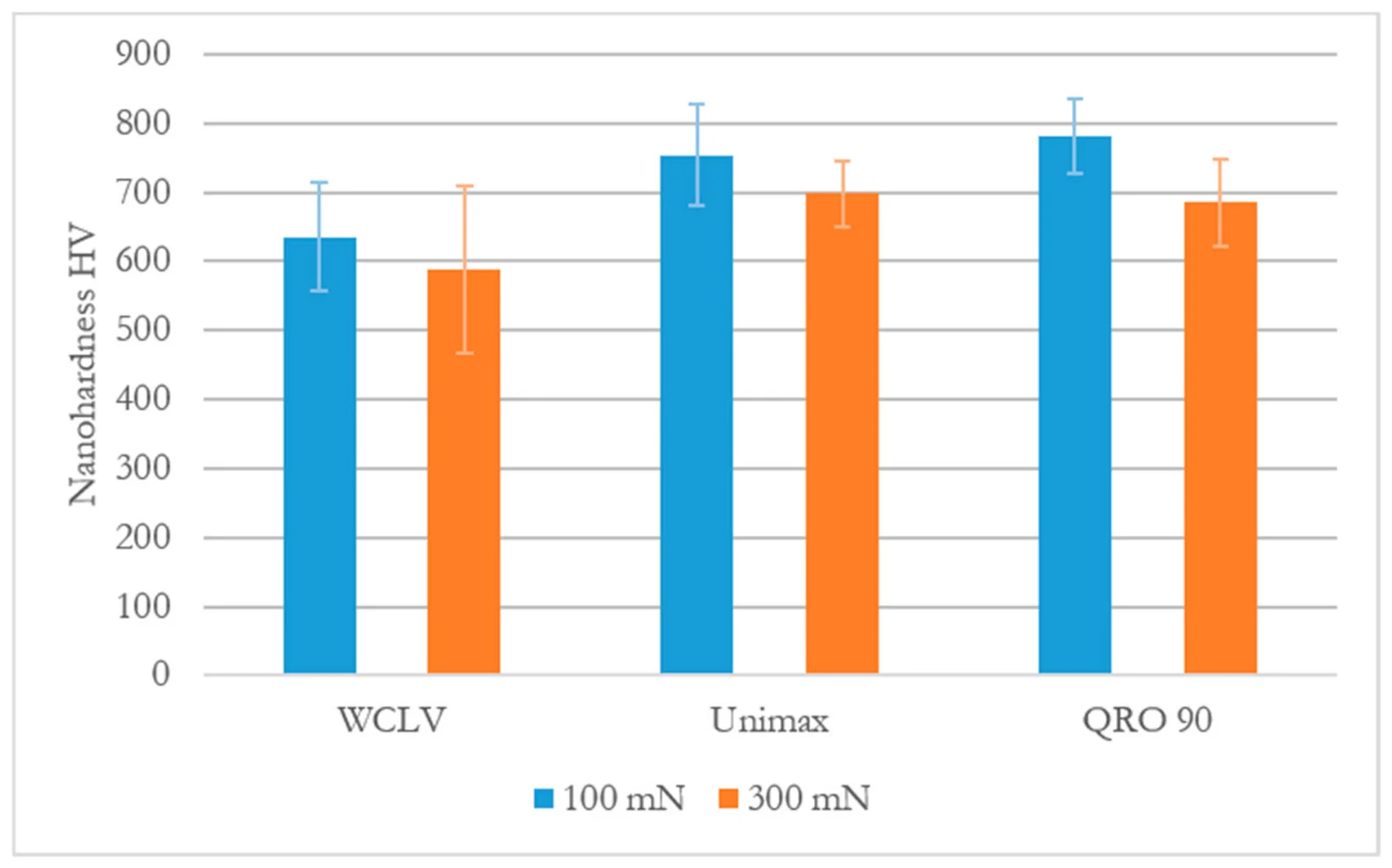
Comparison of Vickers hardness values obtained from nanoindentation tests.

Figure 4 presents the Vickers hardness values obtained from nanoindentation tests performed at loads of 100 mN and 300 mN.

At a load of 100 mN, the highest hardness was recorded for Uddeholm QRO 90 Supreme, reaching 781.22 ± 53.31 HV. A slightly lower hardness was observed for Uddeholm Unimax (753.79 ± 73.54 HV), while WCLV exhibited the lowest value of 635.21 ± 78.05 HV. Increasing the load to 300 mN resulted in a reduction in hardness for all investigated materials. Under these conditions, Uddeholm Unimax exhibited the highest hardness of 698.89 ± 47.66 HV, followed by Uddeholm QRO 90 Supreme with 684.97 ± 62.78 HV. WCLV again showed the lowest hardness, equal to 588.24 ± 122.26 HV.

The decrease in hardness with increasing load may be associated with the indentation size effect, which is commonly observed in nanoindentation measurements. The reduction was particularly pronounced for Uddeholm QRO 90 Supreme, whose hardness decreased by approximately 12.3%, while Uddeholm Unimax and WCLV showed reductions of approximately 7.3% and 7.4%, respectively.

Table 2 presents the indentation elastic modulus (EIT) values determined for the investigated steels at loads of 100 mN and 300 mN.

At a load of 100 mN, the highest indentation modulus was measured for Uddeholm Unimax, reaching 256.62 ± 8.56 GPa. Similar values were obtained for Uddeholm QRO 90 Supreme (249.31 ± 12.53 GPa) and WCLV (247.30 ± 18.20 GPa). At the higher load of 300 mN, the elastic modulus values decreased slightly for all materials. WCLV exhibited an EIT of 246.05 ± 37.19 GPa, while Uddeholm QRO 90 Supreme reached 243.99 ± 14.09 GPa. The largest reduction was observed for Uddeholm Unimax, whose modulus decreased to 232.93 ± 11.44 GPa.

In general, the investigated steels exhibited relatively similar indentation modulus values, ranging from approximately 233 to 257 GPa. Compared with hardness, the differences in EIT were less pronounced, indicating that the elastic response of the investigated materials was broadly comparable despite noticeable differences in resistance to plastic deformation.

Overall, the nanoindentation results confirm the trends observed during conventional hardness measurements and tribological testing. Uddeholm Unimax and Uddeholm QRO 90 Supreme exhibited superior local mechanical properties compared with WCLV steel. The higher hardness values obtained for Uddeholm Unimax and Uddeholm QRO 90 Supreme are consistent with their improved wear resistance observed in the ball-on-disc tests and industrial forging trials. Furthermore, the relatively high elastic modulus values indicate good resistance to elastic deformation under localized contact loading, which is beneficial for hot-forging tool applications.

### 3.3. Chemical Composition Analysis

**Figure 5:**
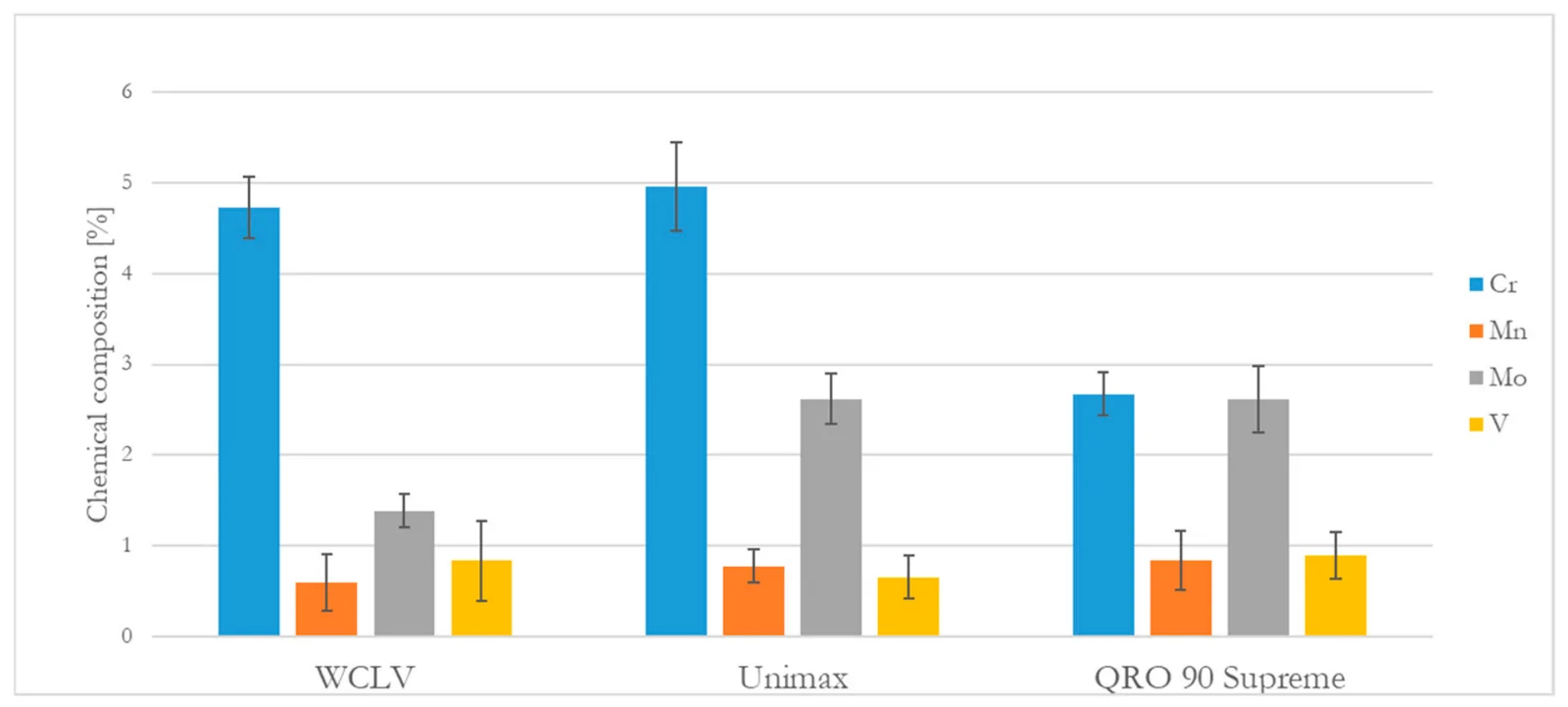
Comparison of the chemical compositions (wt.%) of the investigated materials [1].

The differences in hardness observed for the investigated steels can be attributed primarily to variations in their alloying element content. The results of the X-ray fluorescence (XRF) analysis are shown in Figure 5.

An analysis of the chemical composition indicates that the most pronounced differences between the steels are related to chromium and molybdenum content. WCLV and Uddeholm Unimax steels contain comparable amounts of chromium, 4.733% and 4.955%, respectively, which is nearly twice the chromium content measured in Uddeholm QRO 90 Supreme steel (2.672%). Uddeholm QRO 90 Supreme steel exhibits a slightly higher manganese content (0.845%) compared to Uddeholm Unimax (0.777%) and WCLV (0.596%).

More significant differences were observed for molybdenum. WCLV steel contains only 1.386% Mo, whereas both Uddeholm Unimax and Uddeholm QRO 90 Supreme steels exhibit nearly double this amount, at approximately 2.6%. The lowest vanadium content was measured in Uddeholm Unimax steel (0.654%), followed by WCLV (0.835%), while Uddeholm QRO 90 Supreme contained the highest vanadium content (0.895%).

A comparison of chemical composition with hardness values indicates that chromium and molybdenum play a key role in determining the hardness of the investigated steels. In particular, the higher molybdenum content appears to exert a stronger influence on hardness than chromium [30]. Since hardness is directly related to resistance against abrasive wear, these findings confirm that alloying strategy significantly affects tool durability.

### 3.4. Tribological Behavior and Friction Coefficient

Ball-on-disc wear tests were conducted at two contact radii, r = 19 mm and r = 22 mm. For the smaller radius, the linear sliding speed was 11.938 mm/min with a friction path of 119.38 m, while for the larger radius, the linear speed increased to 13.823 mm/min and the friction path to 138.23 m.

**Figure 6:**
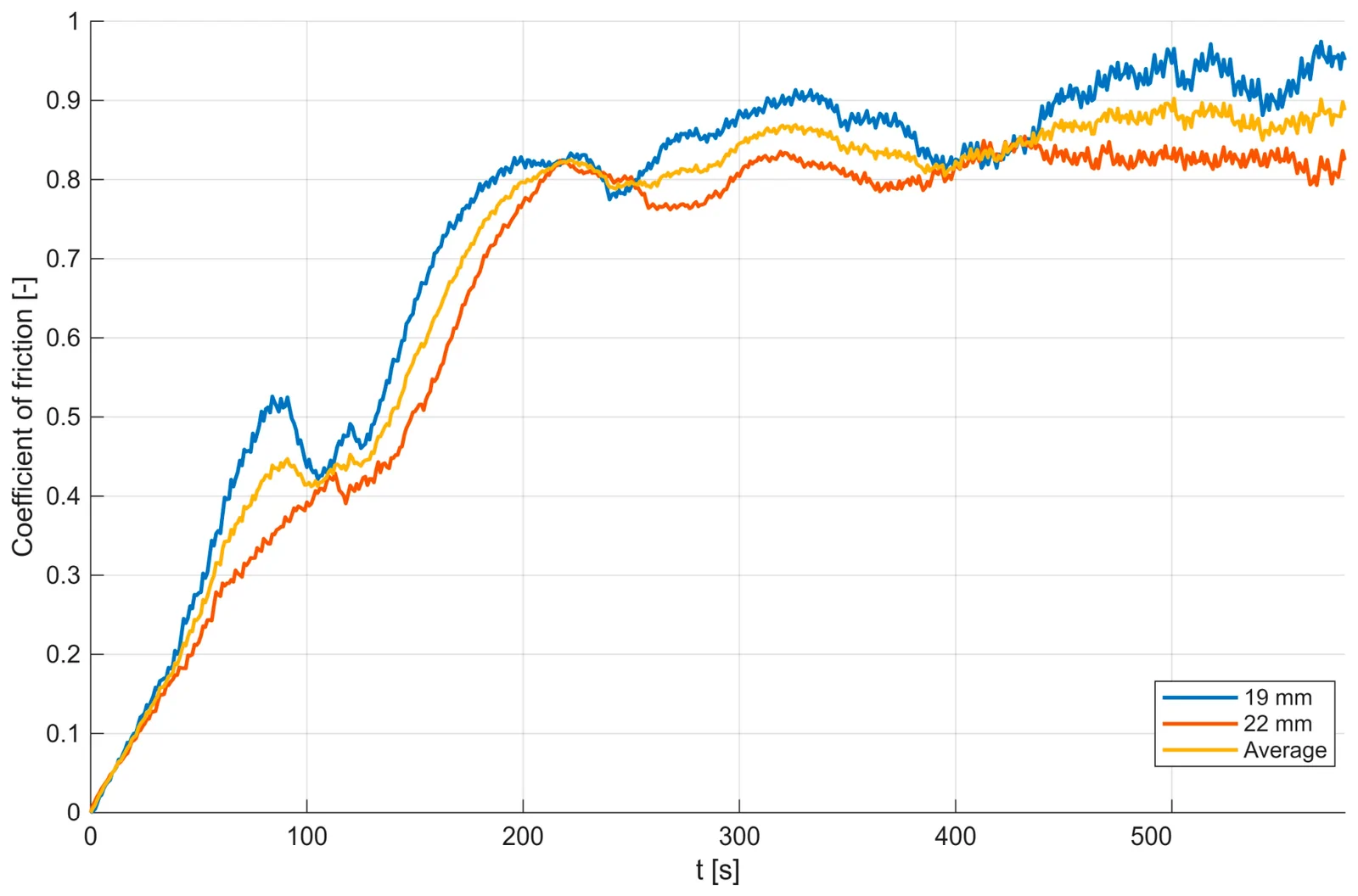
Evolution of the friction coefficient of the WCLV steel as a function of time [1].

The evolution of the friction coefficient over time for WCLV steel is shown in Figure 6.

An initial running-in phase characterized by a rapid increase in the friction coefficient was observed, followed by stabilization. The stabilized friction coefficient reached approximately 0.92 for r = 19 mm and 0.82 for r = 22 mm, resulting in an average value of 0.87.

**Figure 7:**
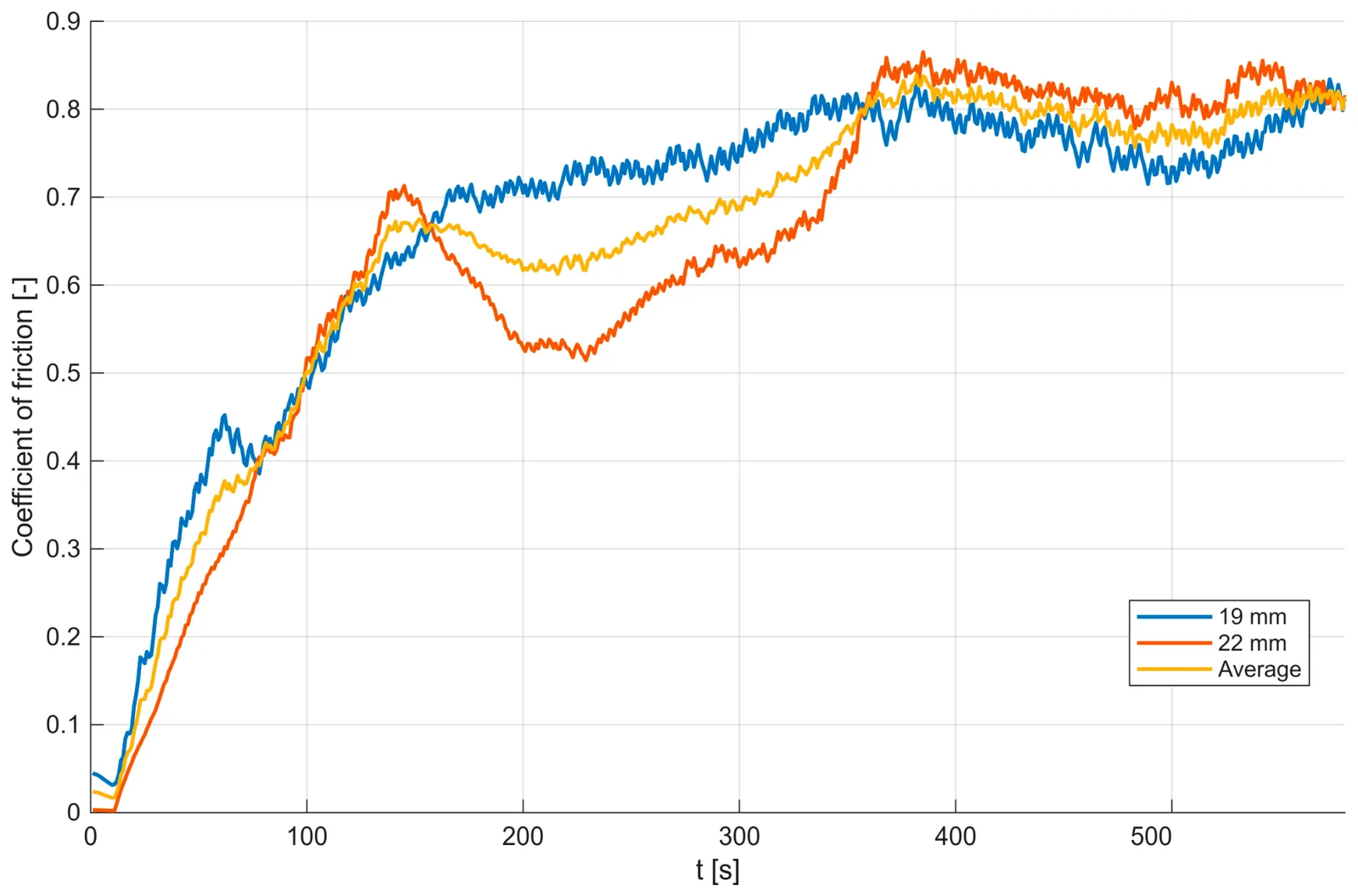
Time-dependent evolution of the friction coefficient of the Uddeholm Unimax steel [1].

The corresponding results for Uddeholm Unimax steel are presented in Figure 7.

A similar running-in behavior was observed, followed by stable friction conditions. However, the friction coefficient values were lower, reaching 0.80 for r = 19 mm and 0.76 for r = 22 mm, with an average value of 0.78.

**Figure 8:**
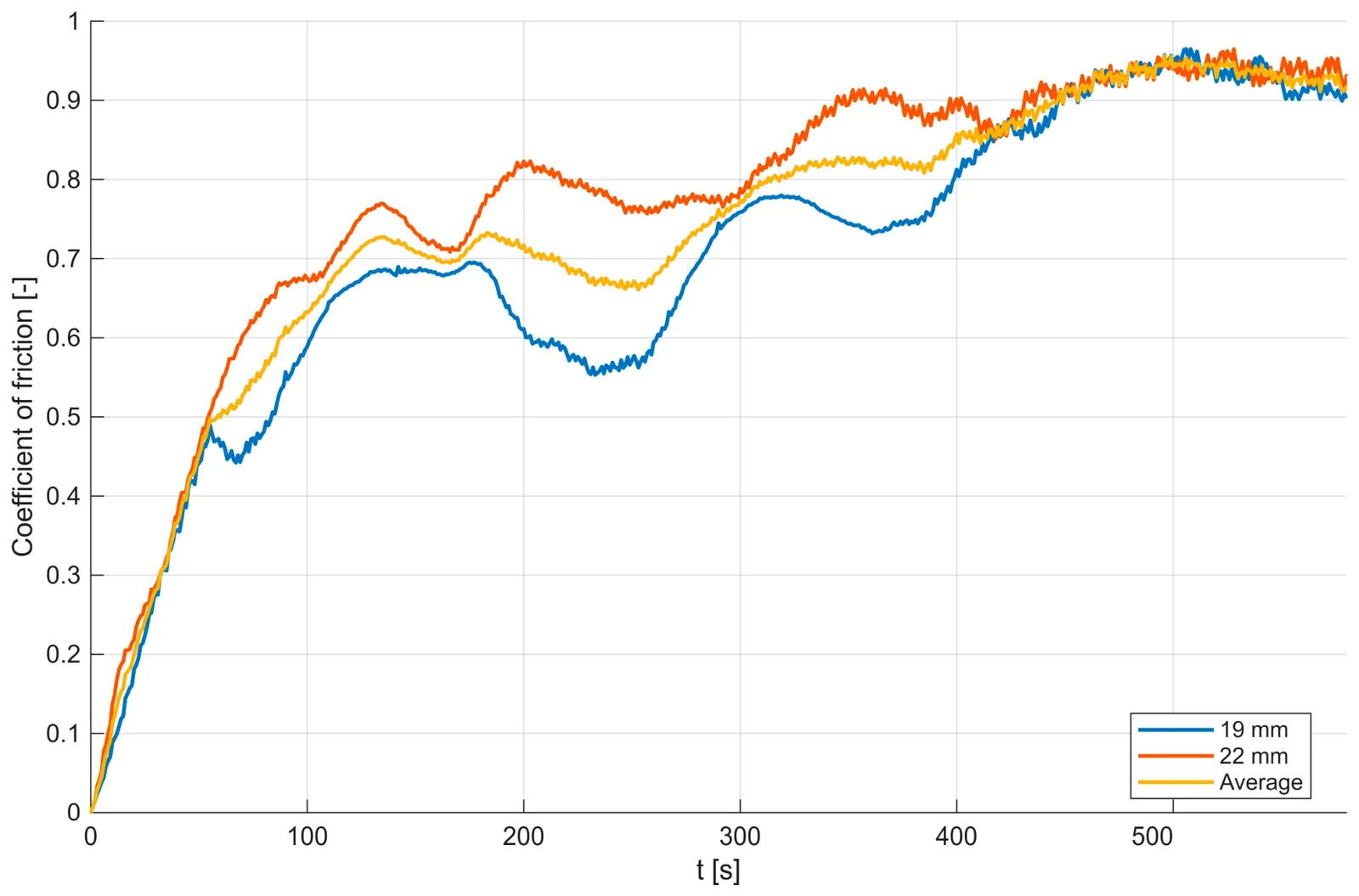
Evolution of the friction coefficient of the Uddeholm QRO 90 Supreme steel as a function of time [1].

The friction coefficient evolution for Uddeholm QRO 90 Supreme steel is shown in Figure 8.

After the running-in phase, the friction coefficient stabilized after approximately 450 s. The stabilized values for both radii were nearly identical, at approximately 0.91.

**Figure 9:**
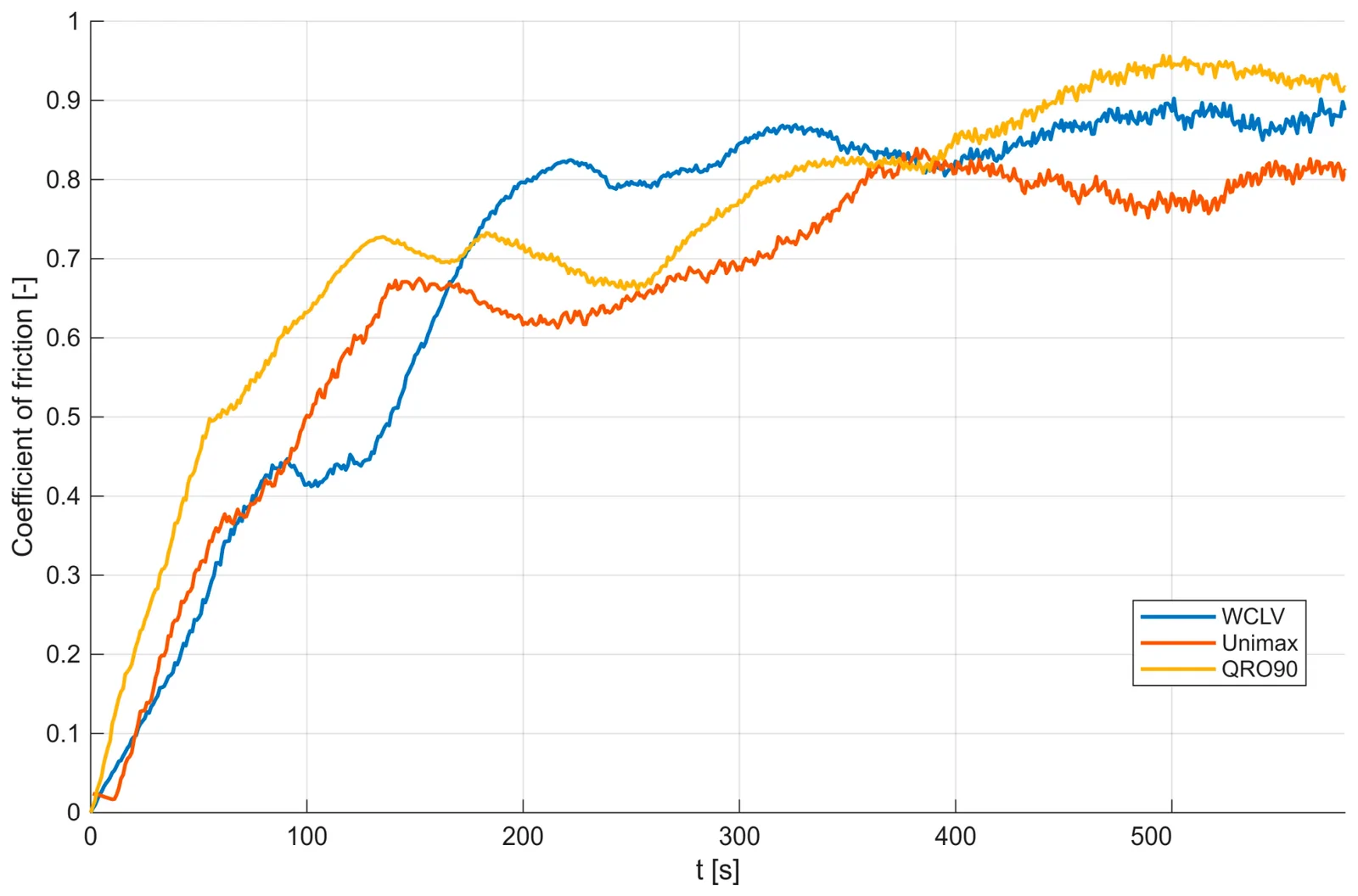
Comparison of friction coefficient–time curves for the tested tool steels [1].

A comparative overview of the average friction coefficients for all materials is provided in Figure 9.

**Figure 10:**
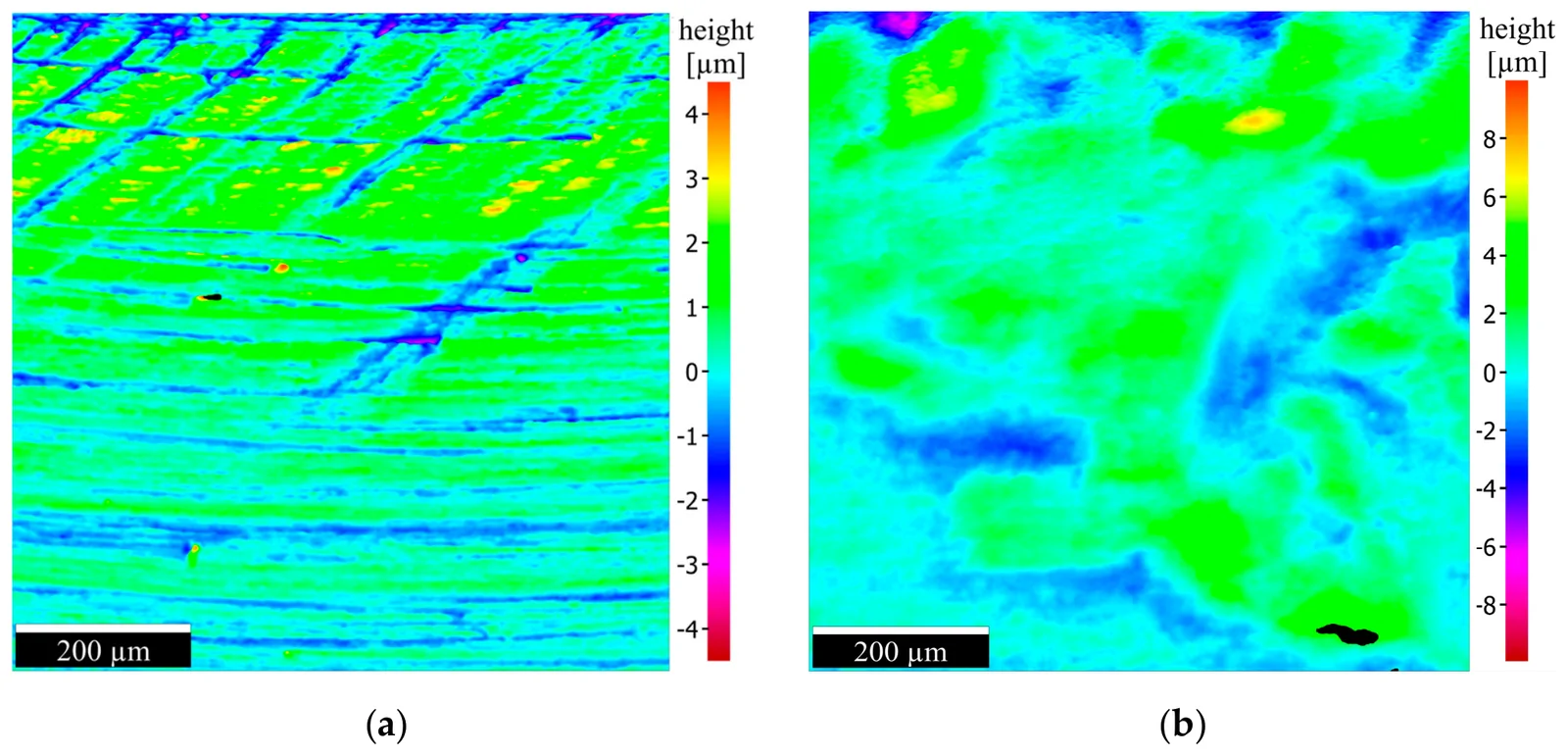
Surface topography of the WCLV steel punch: (**a**) before forging; (**b**) after forging [1].

During the running-in stage (0–200 s), a rapid increase in CoF is observed across all variants, resulting from the geometric adjustment of the contact surfaces and the removal of primary oxide layers. As shown in Figure 7, Figure 8, Figure 9 and Figure 10, the stabilized friction coefficient values reached a high range of 0.81–0.98. The highest friction coefficient was observed for Uddeholm QRO 90 Supreme steel, followed by WCLV steel, while Uddeholm Unimax steel exhibited the lowest friction coefficient. Since lower friction coefficients are generally associated with reduced wear intensity, these results indicate superior tribological performance of Uddeholm Unimax steel.

Such elevated values are indicative of severe adhesive interaction and intense metallurgical bonding (galling), which is typical for unlubricated steel-on-steel contacts under high contact pressures. After approximately 200 s, the friction curves enter a steady-state regime characterized by relatively stable CoF values with minor fluctuations. These oscillations are commonly associated with the cyclic formation and removal of adhesive junctions and transfer layers on the contact surfaces. During sliding, fragments of material may adhere to the counter-body and subsequently detach, producing repeated adhesion–delamination events. This process leads to intermittent variations in friction and contributes to the generation of wear debris.

The final stabilization of the friction coefficient occurs from approx. 350 s for WCLV and Uddeholm Unimax steel and approx. 450 s for Uddeholm QRO 90 Supreme steel. The persistence of high friction levels throughout the test suggests that adhesive wear remains the dominant wear mechanism, accompanied by secondary abrasive effects caused by detached particles trapped within the contact zone. Such debris may locally increase plowing and micro-cutting, further contributing to the observed friction fluctuations and material removal [31,32,33].

### 3.5. Volumetric Wear Analysis

After tribological testing, wear tracks were analyzed using optical profilometry. The cross-sectional areas of the wear scars were measured at three locations for each test, allowing for calculation of volumetric wear. A comparison of the volumetric wear values is presented in Table 3.

The lowest volumetric wear was recorded for Uddeholm Unimax steel (0.0807 ± 0.0167 mm^3^), confirming its superior resistance to material removal under the applied test conditions. WCLV and Uddeholm QRO 90 Supreme exhibited higher wear values of 0.2174 ± 0.0520 mm^3^ and 0.2323 ± 0.0555 mm^3^, respectively. Although the difference between WCLV and QRO 90 Supreme was limited, both materials showed substantially greater wear than Uddeholm Unimax.

### 3.6. Surface Topography and Roughness Evolution After Industrial Forging

Figure 10 illustrates the surface topography of the punch manufactured from WCLV steel before and after the forging process.

A pronounced transformation of the surface morphology can be observed after forging 16,250 components. The initial machining marks visible on the punch surface before operation disappear and are replaced by a highly irregular topography, indicating substantial material removal and surface degradation during service.

The quantitative assessment of surface condition changes is summarized in Table 4.

After forging, an increase in roughness parameters S_a_, S_q_, and S_10z_ is recorded, confirming intensified surface roughening. Additionally, the parameters V_mc_ and V_vc_ increase, reflecting greater profile heights and deeper surface features. In contrast, the kurtosis S_ku_ and the V_vc_/V_mc_ ratio remain nearly unchanged, suggesting that although the amplitude of surface deviations increases, the statistical distribution of surface heights is preserved.

**Figure 11:**
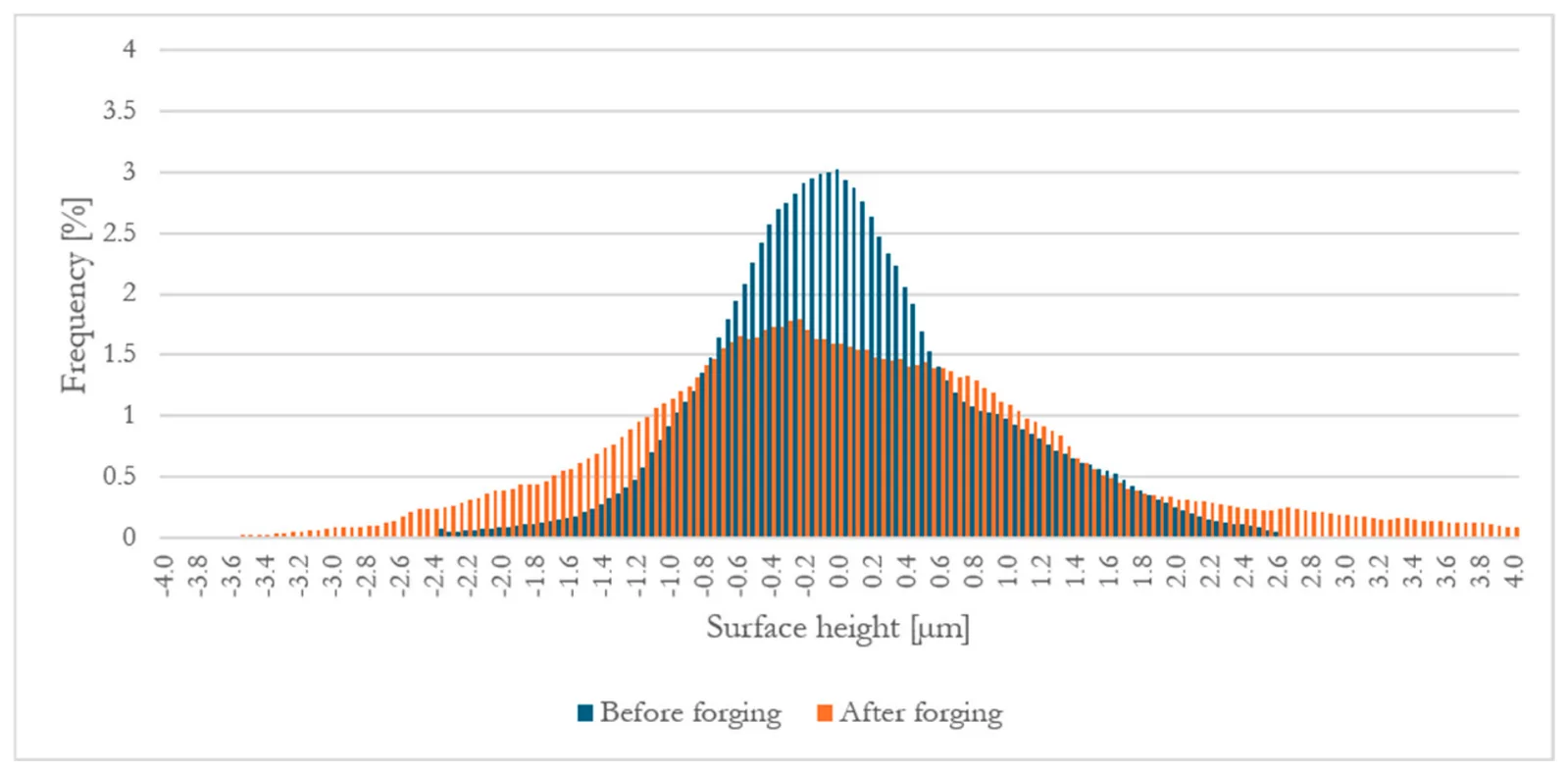
Histogram of surface height distributions for WCLV steel punch [1].

**Figure 12:**
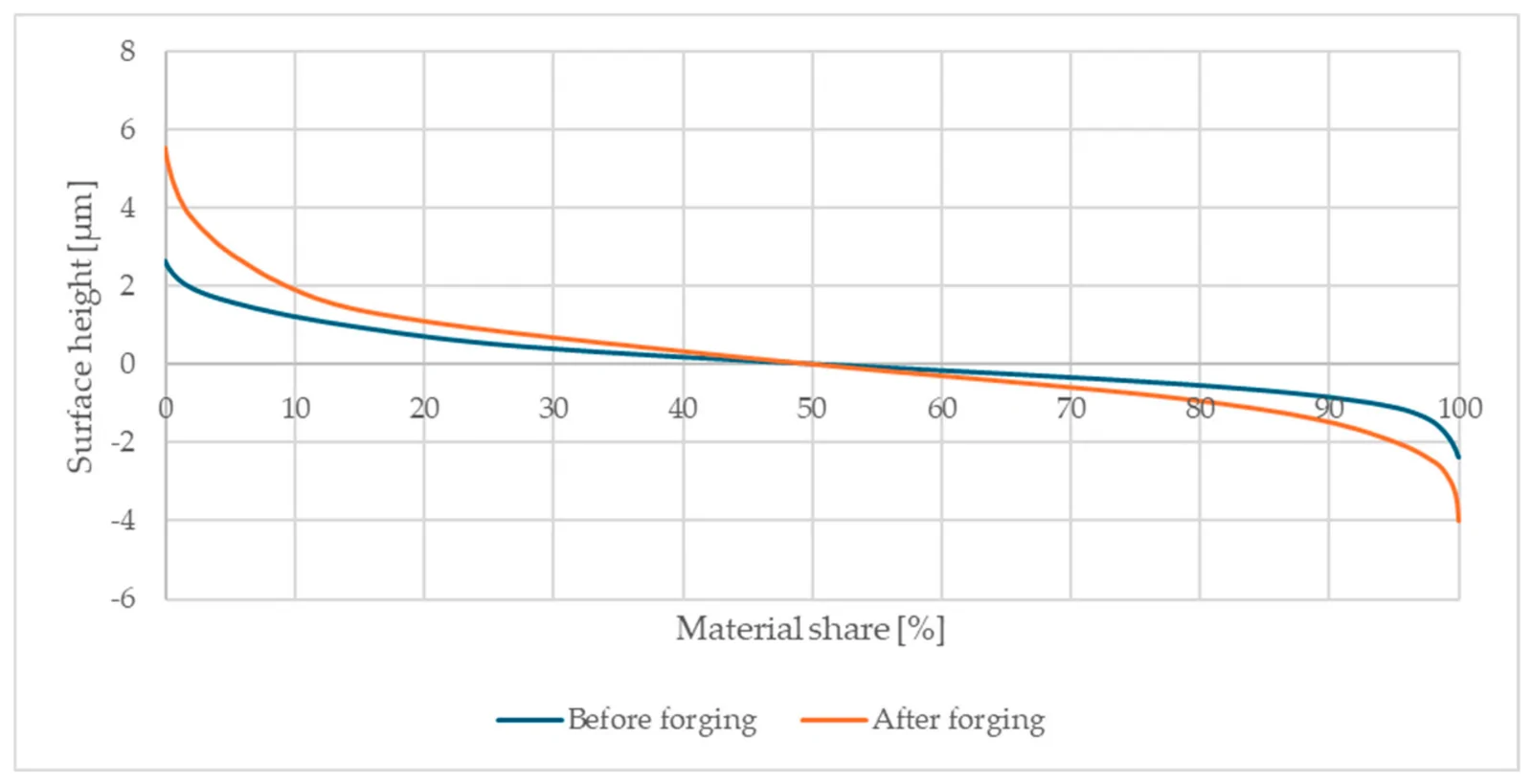
Surface roughness profile curves obtained for the WCLV steel punch [1].

To further illustrate the evolution of the surface profile, histograms of surface height distribution and material ratio curves were generated, as shown in Figure 11 and Figure 12, respectively.

These results indicate progressive wear manifested by the formation of pronounced peaks and deep valleys. While deeper valleys may enhance lubricant retention, the emergence of high asperities increases the susceptibility of the surface to further abrasive and adhesive wear.

**Figure 13:**
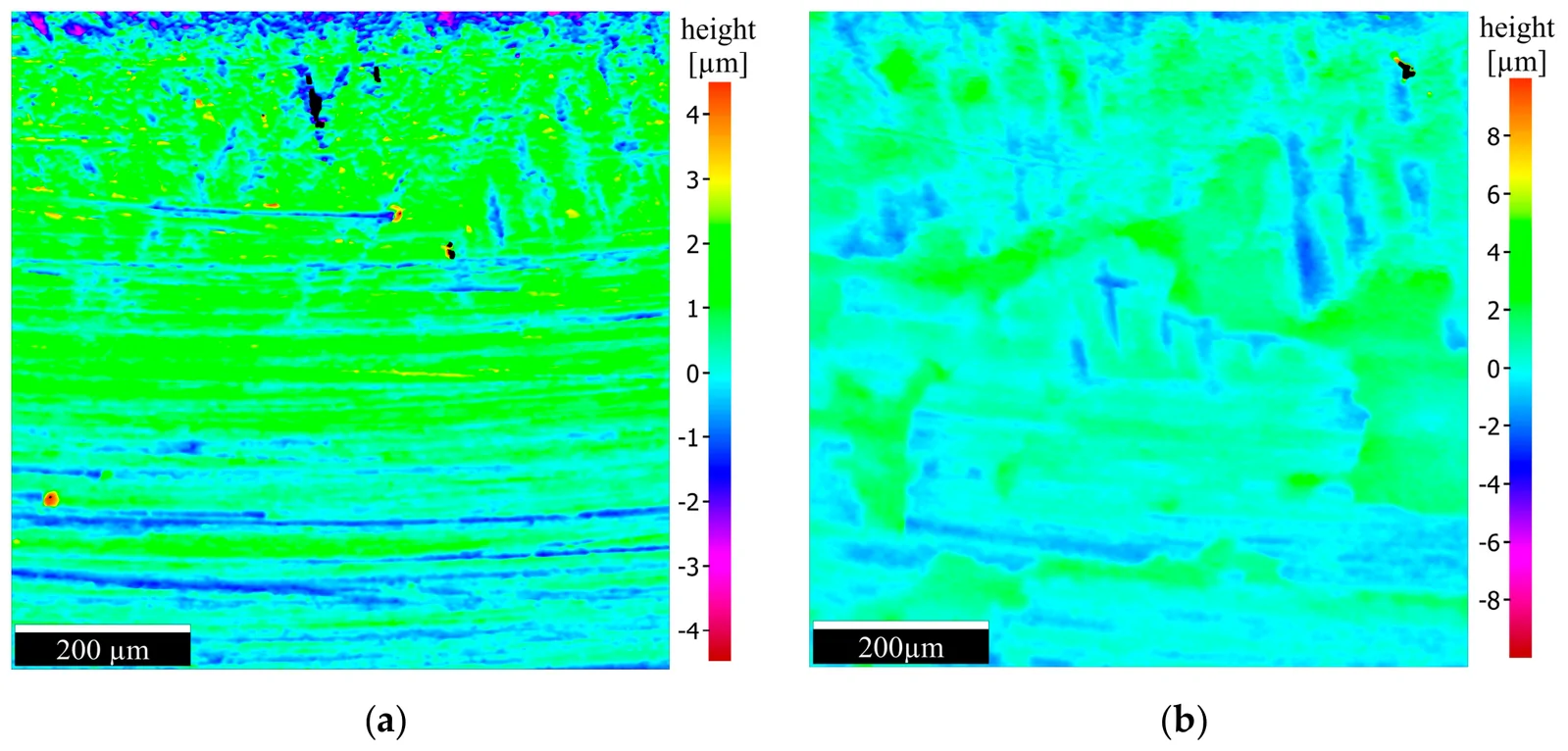
Surface topography of the Uddeholm Unimax steel punch: (**a**) before forging; (**b**) after forging [1].

Figure 13 presents the surface topography of the Uddeholm Unimax steel punch before and after forging.

In contrast to WCLV steel, Uddeholm Unimax exhibits noticeable surface smoothing after forging. The post-forging surface is characterized by relatively small deviations, indicating limited material degradation.

The corresponding roughness parameters are listed in Table 5.

A reduction in S_a_ and S_q_ values is observed, while S_10z_ increases, suggesting the presence of isolated high asperities occupying a small fraction of the surface area. The increase in the V_vc_/V_mc_ ratio and kurtosis S_ku_ indicates a surface dominated by minor height deviations, confirming a different wear mechanism compared to WCLV steel.

**Figure 14:**
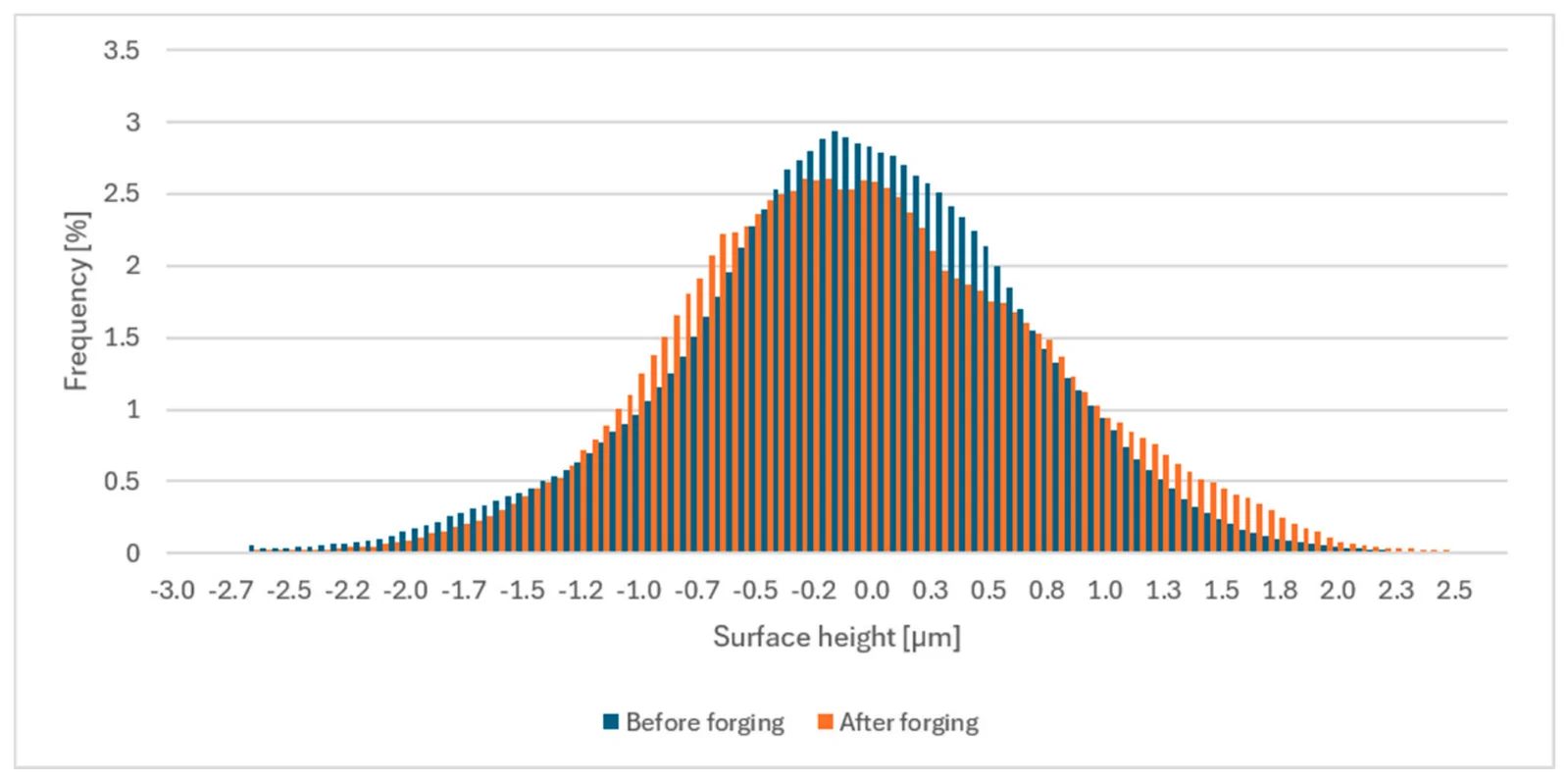
Histogram of surface height distribution for the Uddeholm Unimax steel punch [1].

**Figure 15:**
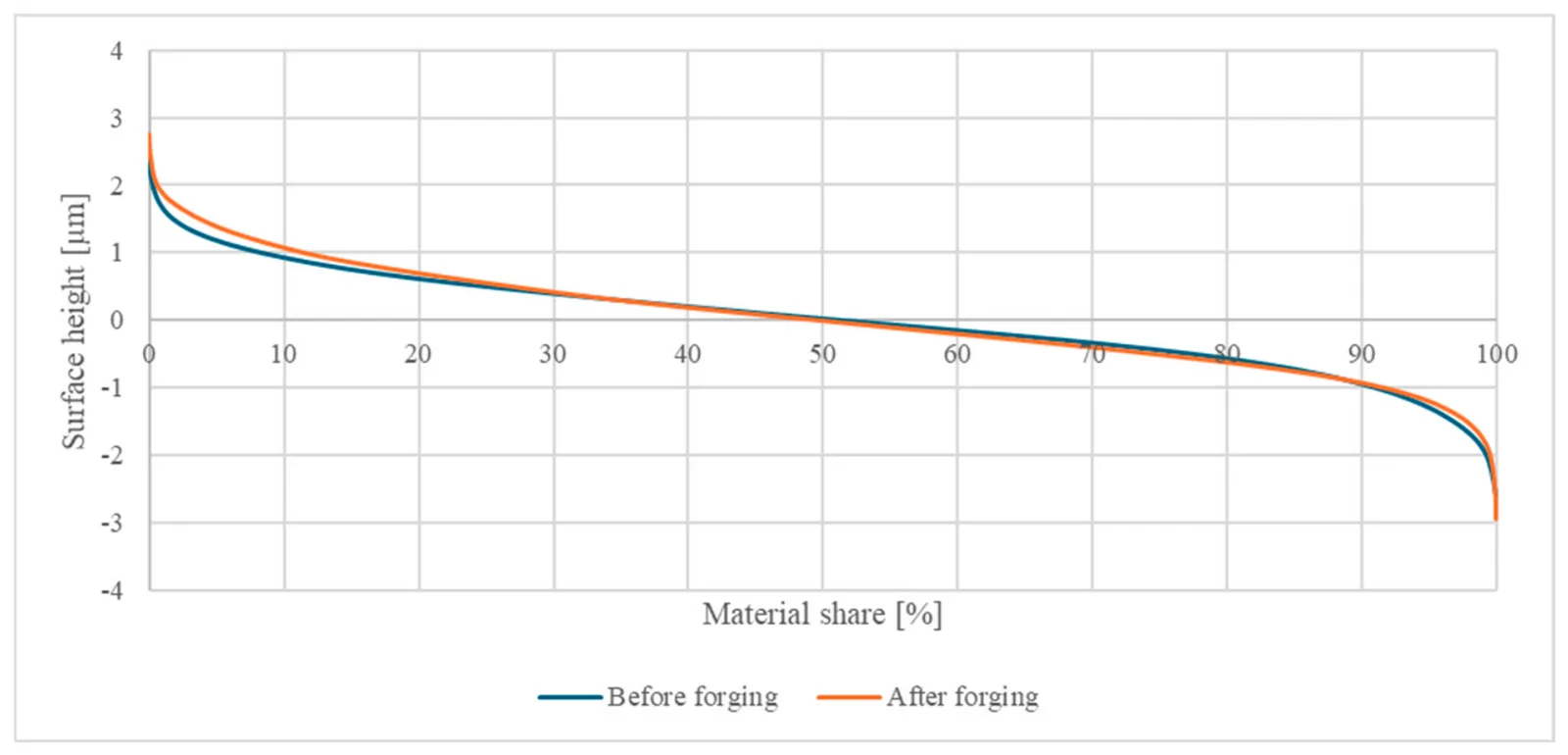
Surface roughness profile curves obtained for the Uddeholm Unimax steel punch [1].

The surface height histograms and material ratio curves for Uddeholm Unimax steel are shown in Figure 14 and Figure 15.

The high similarity of the distributions before and after forging confirms minimal wear and excellent surface stability of Uddeholm Unimax steel.

**Figure 16:**
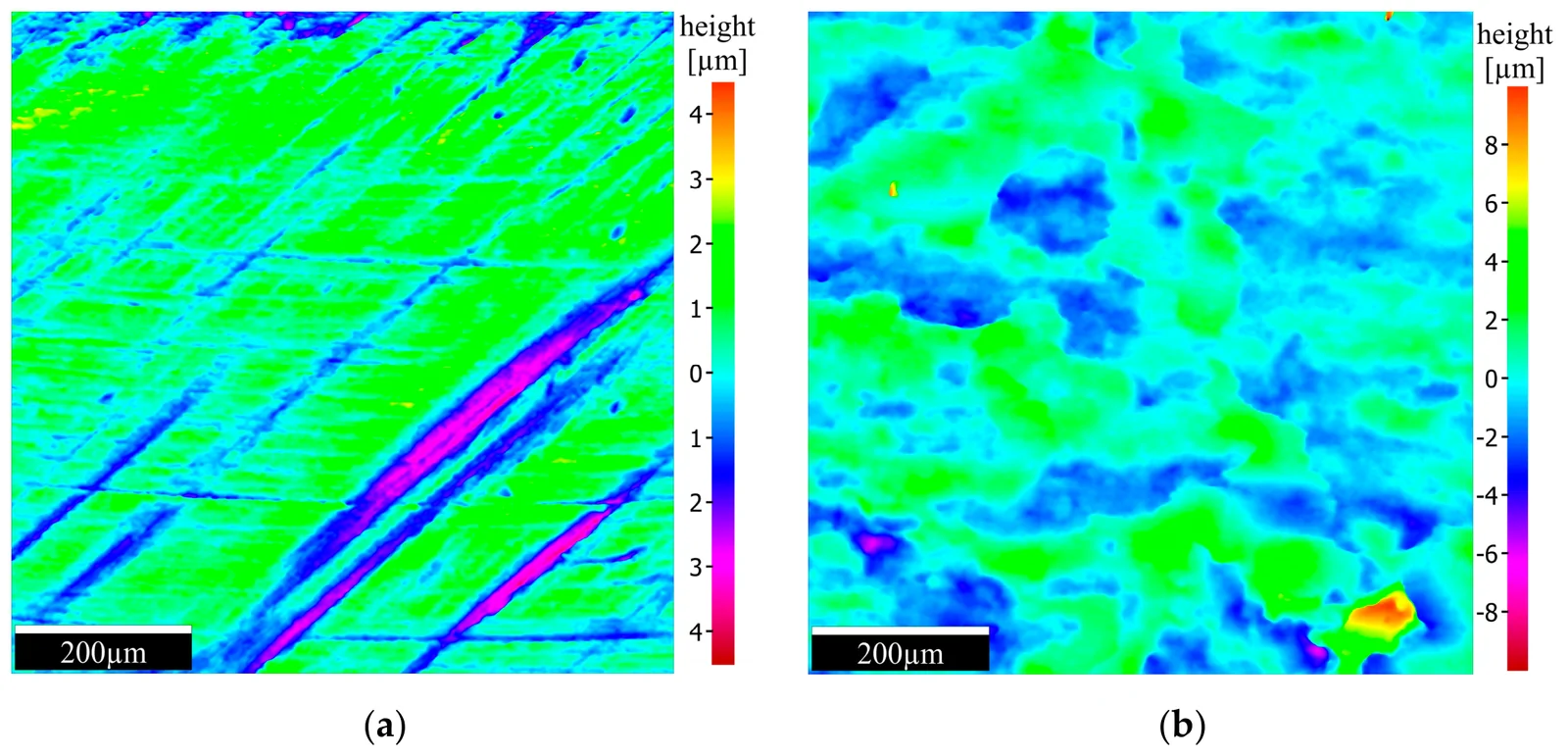
Surface topography of the Uddeholm QRO 90 Supreme steel punch: (**a**) before forging; (**b**) after forging [1].

Figure 16 shows the surface topography of the Uddeholm QRO 90 Supreme steel punch before and after forging.

After forging, the surface becomes highly irregular, with numerous elevations and depressions, indicating advanced wear. Deep scratches visible on the initial surface are attributed to handling or transport damage rather than operational wear.

The surface roughness parameters for Uddeholm QRO 90 Supreme steel are summarized in Table 6.

An increase in S_a_ and S_10z_ values is observed, accompanied by a 12% rise in the V_vc_/V_mc_ ratio, which indicates a reduced material fraction in the core zone and diminished load-bearing capacity.

**Figure 17:**
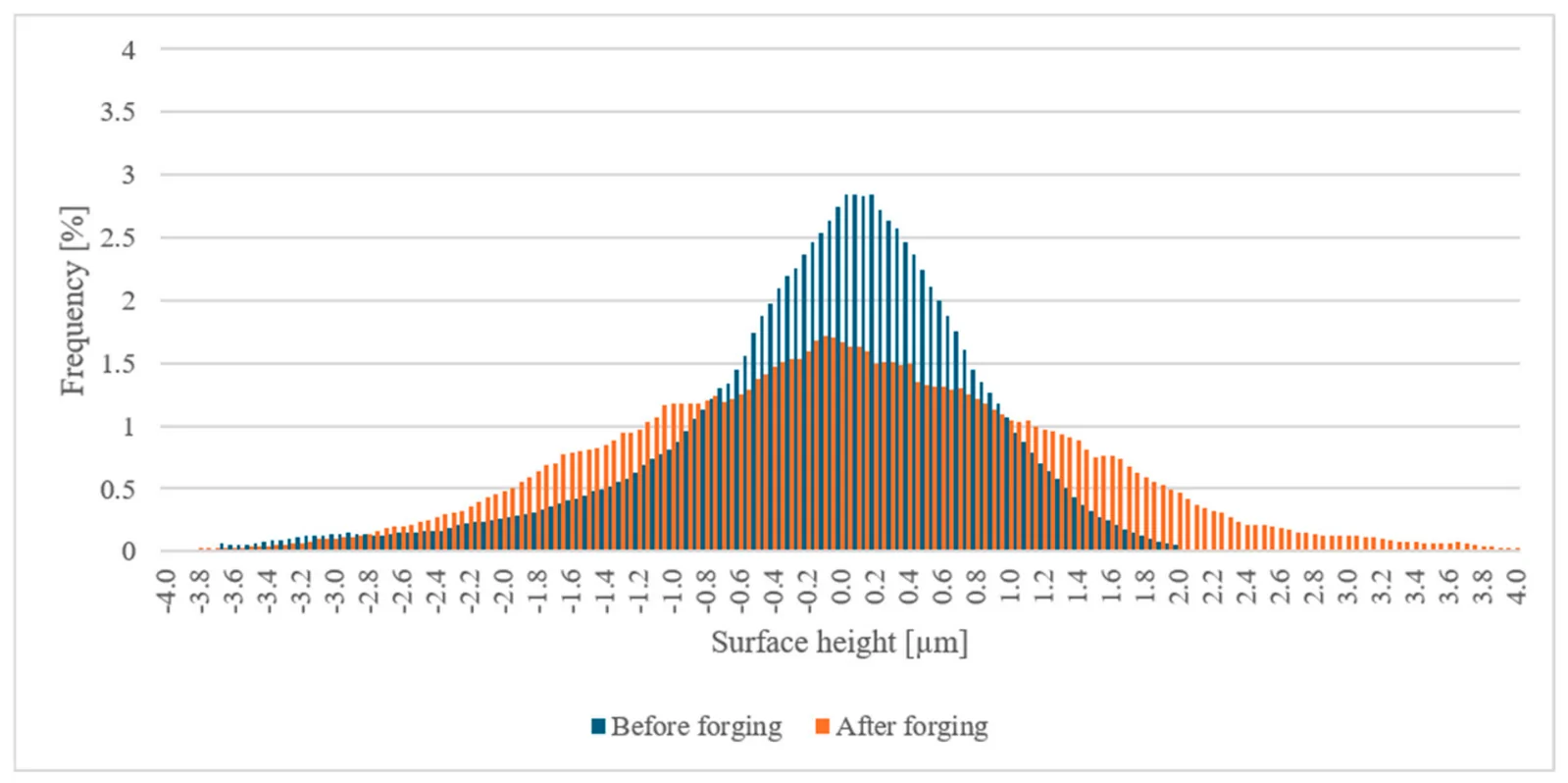
Histogram of surface height distributions for Uddeholm QRO 90 Supreme steel punch [1].

**Figure 18:**
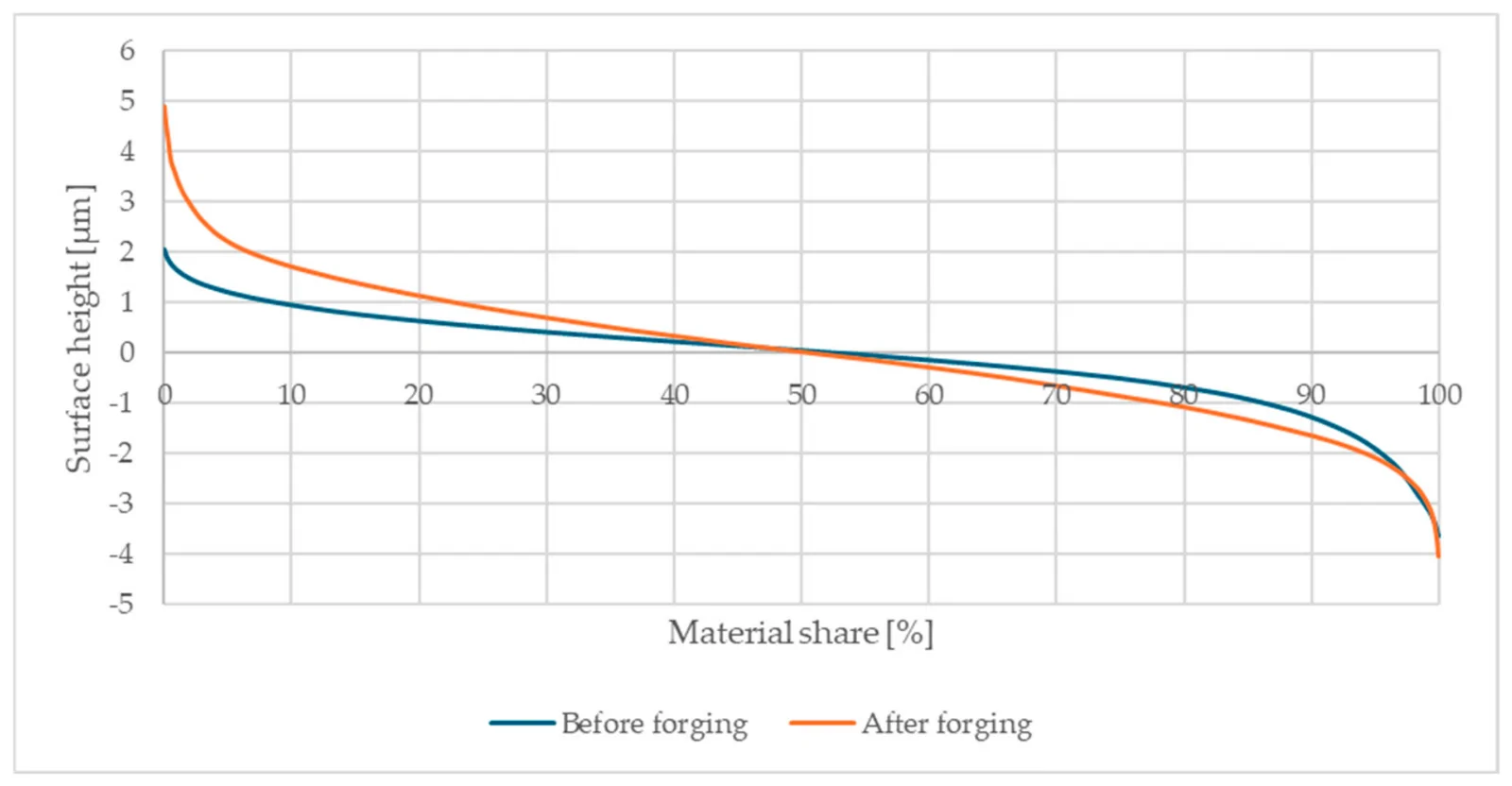
Surface roughness profile curves obtained for the Uddeholm QRO 90 Supreme steel punch [1].

The evolution of surface height distributions and material ratio curves for Uddeholm QRO 90 Supreme steel is presented in Figure 17 and Figure 18.

These results confirm progressive surface degradation and increased roughness after forging, consistent with the observed wear patterns.

### 3.7. Comparative Evaluation of Tool Steels After Forging

A direct comparison of the surface roughness parameters measured on punches manufactured from the investigated tool steels after forging is provided in Table 7.

The data clearly indicate that Uddeholm Unimax steel exhibits the most favorable surface condition after service. In particular, the S_q_ parameter reaches the lowest value (0.951), reflecting minimal surface irregularities and superior resistance to wear. Additionally, this steel is characterized by the lowest kurtosis (S_ku_), which indicates a high proportion of surface features with small height deviations and the absence of pronounced asperities. Such a surface morphology is beneficial for load distribution and reduces the likelihood of localized stress concentrations during forging operations.

**Figure 19:**
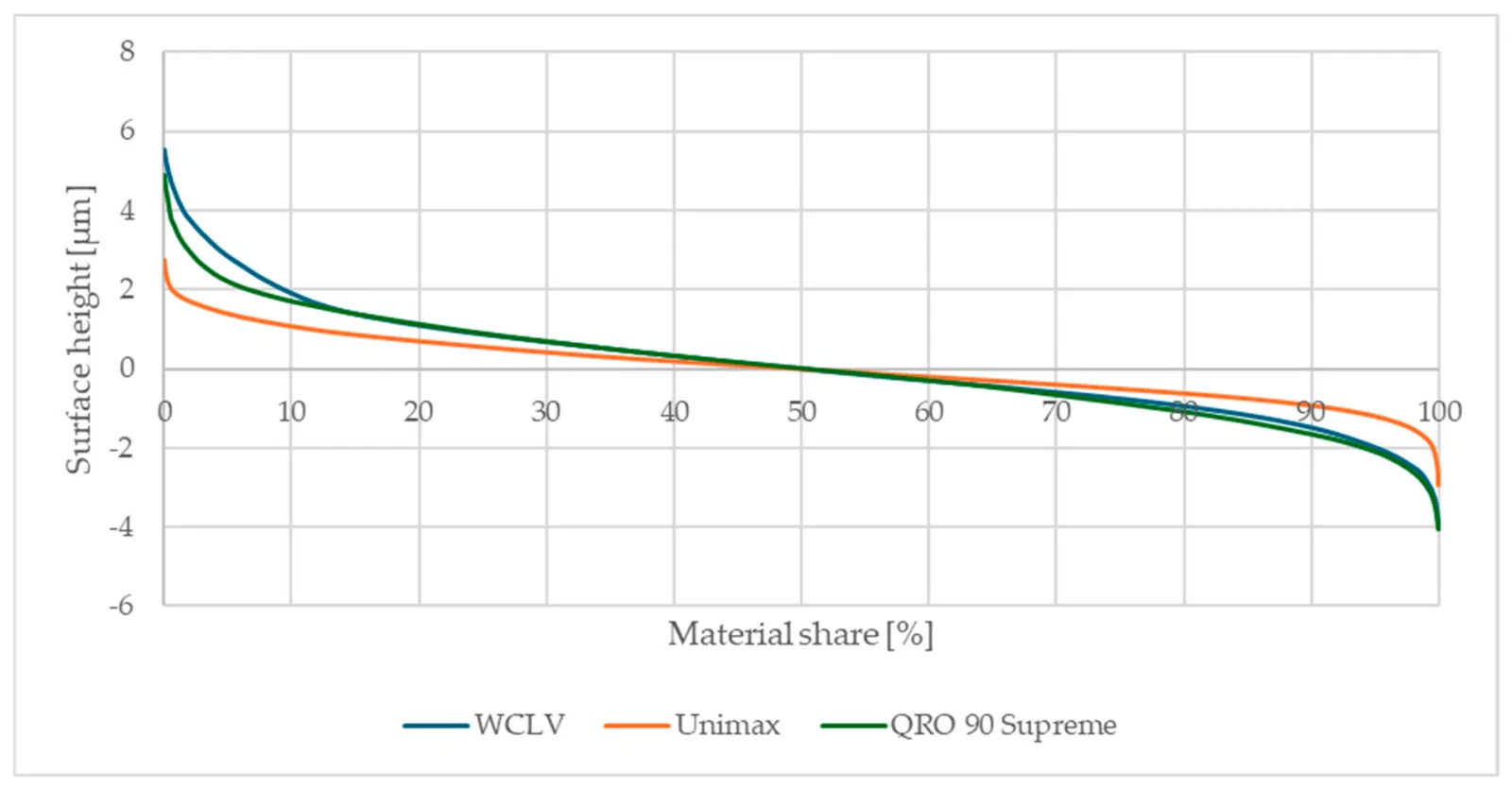
Comparison of surface roughness profile curves for punches made of the tested tool steels [1].

To further illustrate the differences in wear behavior between the tested materials, a comparison of material ratio curves obtained after forging is presented in Figure 19.

The curves reveal that the punch made of Uddeholm Unimax steel possesses the lowest overall profile height and the highest load-bearing capacity among the investigated materials. In contrast, punches manufactured from WCLV and Uddeholm QRO 90 Supreme steels show higher profile elevations, indicating more advanced surface degradation. The differences between WCLV and Uddeholm QRO 90 Supreme steels are mainly observed in the region of the highest surface peaks, which are more pronounced for WCLV steel. From a tribological perspective, such elevated asperities increase the susceptibility to abrasive and adhesive wear.

An important aspect of hot forging tool performance is lubrication efficiency, which is strongly influenced by surface topography. Adequate lubricant retention is facilitated by the presence of surface valleys, which act as lubricant reservoirs. All investigated steels exhibit surface features capable of supporting lubrication; however, Uddeholm Unimax steel shows the lowest valley depth. Despite this, its superior hardness and microstructural stability compensate for the reduced lubricant retention, resulting in the overall best wear performance.

### 3.8. Discussion of Alloying Effects and Wear Mechanisms

The results obtained in this study clearly demonstrate that the chemical composition of tool steels plays a decisive role in determining their wear resistance under hot forging conditions. In particular, the combined presence of chromium and molybdenum significantly enhances hardness and resistance to abrasive wear. The superior performance of Uddeholm Unimax steel can be attributed to its optimized alloying concept, characterized by high chromium and molybdenum content, which promotes the formation of stable carbides and improves high-temperature strength.

The observed correlation between molybdenum content and hardness confirms that molybdenum exerts a stronger influence on abrasive wear resistance than chromium alone. While chromium contributes to oxidation resistance and thermal stability, molybdenum effectively strengthens the matrix and delays softening at elevated temperatures. This relationship is reflected in the tribological test results, where Uddeholm Unimax steel exhibits the lowest friction coefficient and minimal volumetric wear.

Additional support for this interpretation is provided by the nanoindentation results. Under a load of 100 mN, Uddeholm QRO 90 Supreme exhibited the highest local hardness (781 HV), followed by Uddeholm Unimax (754 HV) and WCLV (635 HV). However, despite its superior nanoindentation hardness, Uddeholm QRO 90 Supreme did not demonstrate the best tribological performance. In contrast, Uddeholm Unimax combined high nanoindentation hardness with the lowest friction coefficient and the lowest volumetric wear. This observation suggests that tool durability is governed not only by hardness but also by the ability of the material to maintain favorable tribological conditions during sliding contact.

Analysis of the friction behavior during ball-on-disc testing further supports these conclusions. A stable friction coefficient, as observed for Uddeholm Unimax steel, is indicative of a balanced tribological system and limited surface degradation. In contrast, the higher and less stable friction coefficients recorded for WCLV and Uddeholm QRO 90 Supreme steels suggest increased wear intensity and a greater contribution of abrasive and adhesive wear mechanisms.

The surface topography analyses performed after industrial forging provide additional insight into the dominant wear mechanisms. Uddeholm Unimax steel shows limited surface modification, primarily in the form of mild smoothing, whereas WCLV and Uddeholm QRO 90 Supreme steels exhibit significant roughening, deep grooves, and pronounced asperities. These features are characteristic of abrasive wear combined with thermal and mechanical fatigue, which are typical for hot forging tools operating under cyclic loading and high temperatures.

The indentation elastic modulus values obtained from nanoindentation testing were relatively similar for all investigated steels, ranging from approximately 233 to 257 GPa. This indicates that the elastic response of the materials was broadly comparable, whereas the observed differences in wear behavior were primarily associated with resistance to plastic deformation and surface degradation mechanisms rather than elastic stiffness alone.

Despite the valuable insights obtained in this study, several limitations should be acknowledged. First, the investigation was limited to three hot-work tool steels, which restricts the generalizability of the conclusions to a broader range of alloy systems. Second, the ball-on-disc tribological tests represent a simplified model of the complex thermo-mechanical conditions present in industrial hot forging, where contact pressures, temperature gradients, oxidation processes, and lubrication conditions may vary significantly. Third, the industrial validation was based on a limited number of operational observations, and systematic industrial replicates were not available. Finally, although the results were interpreted in relation to alloying composition and expected carbide formation, detailed microstructural validation (e.g., quantitative characterization of carbide distribution or phase analysis after service) was not performed in the present work. Future studies should therefore include a wider range of tool steels, more representative thermo-mechanical testing conditions, repeated industrial trials, and comprehensive microstructural analyses to further elucidate the mechanisms governing tool wear in hot forging processes.

The combined results of conventional hardness testing, nanoindentation, tribological investigations, and industrial forging trials confirm that microstructural stability, controlled carbide distribution, and optimized alloying are critical factors in enhancing the durability of hot forging tools. The superior performance of Uddeholm Unimax steel observed in both laboratory and industrial conditions highlights its suitability for applications requiring extended tool life and reduced maintenance costs. These findings are consistent with previously reported studies [34,35,36,37], which emphasize the importance of alloy design and microstructural optimization in improving the wear resistance and thermal fatigue behavior of hot forging tool steels.

## 4. Conclusions

The comparative study of three hot forging tool steels—WCLV, Uddeholm Unimax, and Uddeholm QRO 90 Supreme—demonstrates the significant influence of material composition and hardness on wear performance under both laboratory and industrial conditions.

- Tribological investigations and nanoindentation measurements demonstrated clear differences in the local mechanical and tribological behavior of the investigated steels. Uddeholm Unimax exhibited the most stable friction coefficient and the lowest volumetric wear, while nanoindentation tests revealed the highest local hardness values for Uddeholm QRO 90 Supreme and Uddeholm Unimax. This behavior was accompanied by the lowest volumetric wear among the tested materials, indicating improved resistance to abrasive and adhesive wear mechanisms under the applied test conditions.
- Surface topography and roughness analyses of punches after industrial forging confirmed that Uddeholm Unimax experienced the smallest increase in surface roughness, suggesting relatively stable surface behavior during operation. In contrast, WCLV and Uddeholm QRO 90 Supreme steels showed more pronounced surface degradation, characterized by deeper grooves and increased roughness parameters, which are consistent with more intensive wear processes.
- The analysis of chemical composition, conventional hardness, and nanoindentation results indicates that higher contents of alloying elements such as chromium and molybdenum contribute to improved resistance to localized plastic deformation and enhanced wear resistance. Although Uddeholm QRO 90 Supreme exhibited the highest nanoindentation hardness under a load of 100 mN, Uddeholm Unimax demonstrated the most favorable overall combination of hardness, friction behavior, and wear resistance. In particular, Uddeholm Unimax, which exhibited the highest hardness among the investigated steels, demonstrated the most favorable combination of friction behavior and wear resistance.
- The indentation elastic modulus values of all investigated steels were relatively similar, ranging from approximately 233 to 257 GPa. Consequently, differences in wear performance appear to be more strongly related to hardness and microstructural characteristics than to elastic stiffness.
- Under the tested conditions, Uddeholm Unimax can therefore be considered the most promising material for improving the durability of hot forging tools. However, the present study has several limitations, including the relatively small number of investigated materials and the simplified nature of the laboratory tribological test compared with real forging conditions. Therefore, further research should include a broader range of tool steels and additional validation under different forging temperatures, loading conditions, and tool geometries to confirm the general applicability of the observed trends.

## Figures and Tables

**Table 1:** Elemental composition of the Uddeholm Unimax steel (wt.%) [1].

	C	Si	Mn	Cr	Mo	V
WCLV	0.35–0.45	0.80–1.20	0.20–0.50	4.50–5.50	1.20–1.50	0.80–1.10
Unimax	0.50	0.20	0.30	5.00	2.30	0.50
QRO 90	0.38	0.30	0.75	2.60	2.25	0.90

**Table 2:** Indentation elastic modulus (EIT) values determined by nanoindentation for the investigated tool steels (GPa).

Maximum Load	100 mN	300 mN
WCLV	247.30 ± 18.20	246.05 ± 37.19
Unimax	256.62 ± 8.56	232.93 ± 11.44
QRO 90	249.31 ± 12.53	243.99 ± 14.09

**Table 3:** Comparison of the volumetric wear of tested steels.

	Volumetric Wear [mm^3^]
WCLV	0.2174 ± 0.0520
Unimax	0.0807 ± 0.0167
QRO 90	0.2323 ± 0.0555

**Table 4:** Surface roughness parameter values of the WCLV steel punch before and after the forging process [1].

Parameter	$S_{a}$ [µm]	$S_{q}$ [µm]	$S_{10z}$ [µm]	$S_{ku}$	$V_{mc}$ [mL/m^2^]	$V_{vc}$ [mL/m^2^]	$V_{vc}$/$V_{mc}$
Before forging	0.714	0.952	8.399	4.969	0.67	1.104	1.648
After forging	1.18	1.603	13.437	4.75	1.119	1.774	1.585
Change	65%	68%	60%	−4%	67%	61%	−4%

**Table 5:** Surface roughness parameter values of the Uddeholm Unimax steel punch before and after forging [1].

Parameter	$S_{a}$ [µm]	$S_{q}$ [µm]	$S_{10z}$ [µm]	$S_{ku}$	$V_{mc}$ [mL/m^2^]	$V_{vc}$ [mL/m^2^]	$V_{vc}$/$V_{mc}$
Before forging	0.796	0.973	14.760	3.291	0.640	0.859	1.342
After forging	0.705	0.944	18.156	6.092	0.688	1.005	1.461
Change	22%	37%	23%	85%	8%	17%	9%

**Table 6:** Surface roughness parameter values of the Uddeholm QRO 90 Supreme steel punch before and after forging [1].

Parameter	$S_{a}$ [µm]	$S_{q}$ [µm]	$S_{10z}$ [µm]	$S_{ku}$	$V_{mc}$ [mL/m^2^]	$V_{vc}$ [mL/m^2^]	$V_{vc}$/$V_{mc}$
Before forging	0.811	1.005	6.813	2.998	0.742	0.904	1.218
After forging	1.091	1.455	15.248	6.802	1.2	1.631	1.359
Change	35%	45%	124%	127%	62%	80%	12%

**Table 7:** Comparison of surface roughness parameter values for punches manufactured from different tool steels [1].

Parameter	$S_{a}$ [µm]	$S_{q}$ [µm]	$S_{10z}$ [µm]	$S_{ku}$	$V_{mc}$ [mL/m^2^]	$V_{vc}$ [mL/m^2^]	$V_{vc}$/$V_{mc}$
WCLV	1.18	1.603	13.437	4.75	1.119	1.774	1.585
Unimax	0.705	0.944	18.156	6.092	0.688	1.005	1.461
QRO 90 Supreme	1.091	1.455	15.248	6.802	1.2	1.631	1.359

## Data Availability

The original contributions presented in this study are included in the article. Further inquiries can be directed to the corresponding author.
